# A deep-learning algorithm using real-time collected intraoperative vital sign signals for predicting acute kidney injury after major non-cardiac surgeries: A modelling study

**DOI:** 10.1371/journal.pmed.1004566

**Published:** 2025-04-29

**Authors:** Sehoon Park, Soomin Chung, Yisak Kim, Sun-Ah Yang, Soie Kwon, Jeong Min Cho, Min Jae Lee, Eunbyeol Cho, Jiwon Ryu, Sejoong Kim, Jeonghwan Lee, Hyung Jin Yoon, Edward Choi, Kwangsoo Kim, Hajeong Lee

**Affiliations:** 1 Department of Internal Medicine, Seoul National University Hospital, Seoul, Korea; 2 Interdisciplinary Program in Bioengineering, Seoul National University, Seoul, Korea; 3 Department of Radiology, Seoul National University Hospital, Seoul, Korea; 4 Department of Transdisciplinary Medicine, Seoul National University Hospital, Seoul, Korea; 5 Department of Internal Medicine, Chung Ang University Hospital, Seoul, Korea; 6 Department of Internal Medicine, Chung-Ang University Gwangmyeong Hospital, Gwangmyeong, Korea; 7 KAIST: Korea Advanced Institute of Science and Technology, Daejeon, Korea; 8 Department of Internal Medicine, Seoul National University Bundang Hospital, Gyeonggi-do, Korea; 9 Department of Internal Medicine, Seoul National University College of Medicine, Seoul, Korea; 10 Department of Internal Medicine, Seoul National University Boramae Medical Center, Seoul, Korea; 11 Department of Biomedical Engineering, Seoul National University College of Medicine, Seoul, Korea; 12 Department of Medicine, Seoul National University, Seoul, Korea; Royal Derby Hospital, UNITED KINGDOM OF GREAT BRITAIN AND NORTHERN IRELAND

## Abstract

**Background:**

Postoperative acute kidney injury (PO-AKI) prediction models for non-cardiac major surgeries typically rely solely on preoperative clinical characteristics.

**Methods and findings:**

In this study, we developed and externally validated a deep-learning-based model that integrates preoperative data with minute-scale intraoperative vital signs to predict PO-AKI. Using data from three hospitals, we constructed a convolutional neural network-based EfficientNet framework to analyze intraoperative data and created an ensemble model incorporating 103 baseline variables of demographics, medication use, comorbidities, and surgery-related characteristics. Model performance was compared with the conventional SPARK model from a previous study. Among 110,696 patients, 51,345 were included in the development cohort, and 59,351 in the external validation cohorts. The median age of the cohorts was 60, 61, and 66 years, respectively, with males comprising 54.9%, 50.8%, and 42.7% of each cohort. The intraoperative vital sign-based model demonstrated comparable predictive power (AUROC (Area Under the Receiver Operating Characteristic Curve): discovery cohort 0.707, validation cohort 0.637 and 0.607) to preoperative-only models (AUROC: discovery cohort 0.724, validation cohort 0.697 and 0.745). Adding 11 key clinical variables (e.g., age, sex, estimated glomerular filtration rate (eGFR), albuminuria, hyponatremia, hypoalbuminemia, anemia, diabetes, renin-angiotensin-aldosterone inhibitors, emergency surgery, and the estimated surgery time) improved the model’s performance (AUROC: discovery cohort 0.765, validation cohort 0.716 and 0.761). The ensembled deep-learning model integrating both preoperative and intraoperative data achieved the highest predictive accuracy (AUROC: discovery cohort 0.795, validation cohort 0.762 and 0.786), outperforming the conventional SPARK model. The retrospective design in a single-nation cohort with non-inclusion of some potential AKI-associated variables is the main limitation of this study.

**Conclusions:**

This deep-learning-based PO-AKI risk prediction model provides a comprehensive approach to evaluating PO-AKI risk prediction by combining preoperative clinical data with real-time intraoperative vital sign information, offering enhanced predictive performance for better clinical decision-making.

## Author summary

**Why was this study done?:**

- Postoperative acute kidney injury (PO-AKI) is a significant complication in non-cardiac major surgeries, adversely affecting patient outcomes.
- Existing predictive models primarily rely on preoperative clinical data, overlooking the impact of intraoperative factors that are critical to PO-AKI development.
- This study aimed to improve PO-AKI prediction by integrating deep-learning analysis of intraoperative vital signs with preoperative clinical data.

**What did the researchers do and find?:**

- A deep-learning model (DL-IVSS) was developed using minute-scale intraoperative vital sign data to predict PO-AKI risk in non-cardiac surgeries.
- The model demonstrated comparable performance to traditional prediction methods relying solely on preoperative data but improved when combined with preoperative information.
- An ensembled deep-learning model incorporating both preoperative and intraoperative data, achieved superior predictive accuracy compared to the baseline DL-IVSS model.

**What do these findings mean?:**

- Integrating intraoperative vital signs into PO-AKI prediction models can enhance predictive performance, providing clinicians with more accurate risk assessments.
- These findings highlight the importance of real-time intraoperative monitoring in mitigating the risk of PO-AKI through timely interventions.
- Limitations include the retrospective nature of the study, single-nation data, and the imputation of missing intraoperative data, warranting further validation in prospective and multi-national settings.

## Abstract 3

Sehoon Park, Soomin Chung and colleagues develop a deep learning model that combines minute-scale intraoperative vital sign data with preoperative clinical data to improve prediction of postoperative risk of acute kidney injury in non-cardiac surgery.

## Introduction

Postoperative acute kidney injury (PO-AKI) is a critical complication after surgery, significantly impacting patient outcomes. While PO-AKI occurs less frequently following non-cardiac major surgeries than cardiac surgeries, it is associated with substantial morbidity, increased healthcare costs, prolonged hospitalization, and even long-term kidney impairment and mortality [1,2]. Despite these impacts, prediction of PO-AKI in non-cardiac surgeries remains a formidable task due to the multifactorial etiology and the lack of universally accepted predictive models.

The risk of PO-AKI in non-cardiac surgeries is determined by both preoperative factors including baseline kidney function and patients’ underlying comorbidities, hemodynamic alteration or related medication usage [3–5]. Previous studies have made certain efforts to build a valid PO-AKI risk prediction model using diverse approaches, including conventional regression analysis, machine learning, and deep-learning techniques [3,4,6–8]. However, many models lack external validation and generalizability, often providing only moderate accuracy, leading to inconsistent adoption [9]. Importantly, most existing models rely exclusively on preoperative variables to enable early implementation of protective strategies for PO-AKI, even though their accuracy remains suboptimal.

Given the kidney’s susceptibility to hemodynamic fluctuations during surgical procedures, integrating intraoperative vital signs into PO-AKI prediction models is imperative [10,11]. Traditionally, these data have been simplified into summary statistics or predefined thresholds to manage the complexity of large-scale time-series information [5,12–14]. However, recent advancements in deep-learning techniques offer promising solutions for effectively handling real-world monitoring datasets [14].

In the current study, our primary objective was to develop a robust PO-AKI risk prediction model using extensive clinical datasets that include both preoperative factors and intraoperative vital signs recorded at minute intervals. Furthermore, we sought to evaluate the potential improvement in predictive performance achieved by integrating deep-learning-based intraoperative vital sign analysis with preoperative information in the context of non-cardiac major surgeries.

## Methods

### Ethical considerations

This study was conducted as a retrospective analysis without a predefined study protocol. Since this was a retrospective study analyzing existing clinical data, the study was not registered in a clinical trial registry. There was no patient or public involvement in the study design, conduct, reporting, or dissemination plans, as this was a retrospective analysis of existing clinical data. This study was approved by the Institutional Review Boards (IRBs) of the Seoul National University Hospital (IRB No. H-2102-192-1203), and Bundang Seoul National University Hospital (IRB No. B-2107-698-401), and the Seoul Metropolitan Government Boramae Medical Center (IRB No. 20-2021-57). The requirement for informed consent was waived by the attending institutional IRBs because this was a retrospective observational study. This study was conducted in accordance with the principles of the Declaration of Helsinki. This study is reported according to the Transparent Reporting of a Multivariable Prediction Model for Individual Prognosis or Diagnosis with Artificial Intelligence (TRIPOD+AI) statement (S1 Checklist).

### Study design and population

The current study is a multi-center retrospective cohort study incorporating data from three datasets from tertiary referral hospitals in South Korea. First, the developmental cohort included adult major non-cardiac surgery cases from 2004 to 2020 in Seoul National University Hospital. Additionally, two external validation datasets were constructed: external validation cohort 1 (EVC 1) included non-cardiac surgery cases from Seoul National University Bundang Hospital from 2004 to 2021, and external validation cohort 2 (EVC 2) comprised cases from Seoul Metropolitan Government-Seoul National University Boramae Medical Center from 2004 to 2020 [4].

The study included adult patients undergoing non-cardiac major surgeries in the fields of general surgery, orthopedic surgery, obstetric-gynecologic surgery, urologic surgery, and neurosurgery. Major surgeries were defined as those with an operation time exceeding one hour, based on previous studies. For patients with multiple surgeries, only the first surgery was analyzed to avoid confounding effects from prior procedures.

The exclusion criteria mainly considered data availability and excluded non-eligible surgery cases such as surgeries directly affecting kidney function or those with established kidney failure. We excluded patients without electronic health records or intraoperative vital sign information (e.g., systolic and diastolic blood pressure (BP) or heart rate), those without appropriate surgery-related data (e.g., missing surgery time information). Cases without sufficient information to evaluate PO-AKI risk, particularly the 11 variables from the SPARK classification [4] were also excluded. In addition, we excluded patients without baseline or follow-up serum creatinine levels within 7 days after surgery. Surgeries for deceased kidney donation, kidney transplantation, or nephrectomy were excluded due to their direct impact on kidney function. Patients with established end-stage kidney disease, baseline eGFR <  15 mL/min/1.73 m^2^, or a history of KRT were excluded. In addition, as serum creatinine elevation ≥  4 mg/dL itself is a criterion for stage 3 AKI, those with such high serum creatinine values before surgery were excluded. Lastly, as we aimed to use this model within 7 days after surgery when follow-up creatinine levels were measured, patients who died during this period were excluded. However, patients who underwent dialysis within this period were included in the analysis, as dialysis represents a severe AKI event.

### Preoperative clinical characteristics

We collected a total of 103 preoperative clinical characteristics to develop a machine learning-based AKI prediction model and to integrate it with our deep-learning algorithm, which leverages time-series intraoperative vital sign signals (DL-IVSS). The variables included baseline demographic data, medication use, preexisting comorbidities, and surgery-related characteristics. A detailed list of the preoperative characteristics is provided below, and the analysis of missing data for these variables is presented in S1 Table. To handle missing data during model training, we imputed missing values for numerical variables using the mean, while categorical variables with missing values were marked as “NaN” and incorporated into the model using a NaN masking approach. Some variables had a high missing rate (e.g., NYHA), however, the variable was remained as we intended to include as many collectible variables as possible along with the imputation method.

A total of 103 preoperative clinical variables were collected to develop and validate our PO-AKI prediction models. Categorical variables included sex, smoking status, operation details (department, type of admission, operation type, types of anesthesia, American Society of Anesthesiologists (ASA) grade, New York Heart Association (NYHA) grade), and patient-oriented medical history, such as diabetes mellitus, hypertension, kidney disease, heart disease, liver disease, tuberculosis, thyroid disease, asthma, chronic obstructive pulmonary disease (COPD), hematologic disease, neurologic disease, other organ comorbidity, vascular disease, and pregnancy history, diagnosis record of comorbidities including diabetes, hypertension, chronic kidney disease, AKI, coronary artery disease, cardiovascular disease, malignancy, and COPD. Additional categorical variables included medication history of anti-diabetic or anti-hypertensive medications, drug usage history within 14 and 90 days prior to surgery for non-steroidal anti-inflammatory drug (NSAID), diuretics, renin-angiotensin-aldosterone system blockades, aspirin, clopidogrel, ezetimibe, fenofibrate, immunosuppressants, direct oral anti-coagulant, low-molecular weight heparin, statin, steroid, and warfarin. Furthermore, eGFR category and dipstick urine test results for albuminuria and hematuria were included. Numerical variables encompassed demographic and clinical parameters such as age, systolic and diastolic BP, heart rate, height, weight, body mass index, duration of admission before operation, estimated operation duration, hemoglobin, hematocrit, platelet count, white blood cell count, erythrocyte sedimentation rate, neutrophil count, c-reactive protein (CRP), sodium, potassium, chloride, total CO2, blood urea nitrogen, creatinine, estimated glomerular filtration rate (eGFR) calculated by Chronic Kidney Disease Epidemiology Collaboration (CKD-EPI) equation [15], total protein, albumin, total cholesterol, low-density lipoprotein, high-density lipoprotein, triglyceride, calcium, phosphate, uric acid, aspartate aminotransferase, alanine aminotransferase, alkaline phosphatase, hemoglobin A1c, parathyroid hormone, urine protein-to-creatinine ratio, total bilirubin, glucose, and prothrombin time (INR).

### Outcome variables

The primary outcome variable was the occurrence of PO-AKI defined according to the KDIGO serum creatinine level criteria (≥1.5-fold increase or ≥ 0.3 mg/dL increase from the baseline value within 7 days after surgery, in alignment with the Acute Disease Quality Initiative recommendation [16]. Among cases of PO-AKI, we defined critical AKI events as stage 2 AKI (≥2.0-fold increase from the baseline value) or AKI associated with acute mortality or the initiation of dialysis within 90 days postoperatively [17]. The critical AKI outcome was defined in the same manner as the original SPARK study and aimed to capture severe AKI events as well as those linked to significant clinical consequences including mortality or dialysis.

### Intraoperative vital sign data preprocessing

We preprocessed intraoperative vital sign data by removing extreme outliers outside the following reference ranges: 30 mmHg ≤  diastolic BP ≤ 240 mmHg, 50 mmHg ≤  systolic BP ≤ 260 mmHg, and 30 bpm ≤  heart rate ≤ 200 bpm. When available, invasive BP measurements (IDBP and ISBP) were used in preference to non-invasive DBP and SBP measurements. To prepare the data for the deep-learning models [e.g., convolutional neural network (CNN)], we unified the sampling rate as one-minute intervals, using forward filling to address missing time points. This approach reflected real-world clinical practice, as it aligns with what attending surgeons or anesthesiologists observe on vital sign monitors. For stable learning, we first applied min-max normalization to scale the data points. As the length of the operation was diverse, we subsequently applied padding with a value of −0.001 after the end of the operation. This padding value was chosen to avoid overlapping with the normalized data points, where min-max normalization sets the minimum value to 0, and to ensure that the padding value remains reasonably close to the data distribution. The padding method was selected because it showed the highest discriminative power compared to mean padding/expanding or repeating methods, which are commonly used to fill empty datapoints.

### Overall process of PO-AKI prediction models

We developed several models to evaluate the discriminative capacity for predicting PO-AKI. First, we engineered the DL-IVSS algorithm. We then compared its performance against SPARK scores, preoperative clinical factors, and intraoperative vital sign summary-level data. Additionally, we assessed whether incorporating these factors enhanced the discriminative capacity of the DL-IVSS algorithm. For model training, the developmental cohort was split into an 80:20 ratio, with 80% of the data used for training and 20% for internal testing. External validation was conducted using independent datasets to evaluate the generalizability and robustness of the models.

### Data processing for adjoining with the preoperative clinical dataset

The initial deep-learning algorithm was constructed with only including intraoperative vital sign signals (DL-IVSS_only). Next, three levels of additional models were constructed. First, the basic demographic model included age, sex, and baseline eGFR (DL-IVSS_PCFs 3: Preoperative Clinical Features 3). The second model, which included major preoperative characteristics, incorporated the eleven SPARK variables (age, sex, baseline eGFR, dipstick urine albuminuria, hyponatremia, hypoalbuminemia, anemia, underlying diabetes mellitus, preoperative use of renin-angiotensin-aldosterone inhibitors, whether the surgery was an emergent operation, and the estimated surgery time) (DL-IVSS_PCFs 11: Preoperative Clinical Features 11). The third model included summary-level intraoperative vital sign data including length, mean, standard deviation, variability, and other information on systolic and diastolic BP and heart rate (S2 Table) (DL-IVSS_ PCVSFs 28: Preoperative Clinical and Vital Sign Features 28). Finally, a tabular machine learning model (preOp_ML) incorporating 103 preoperative variables was developed and used exclusively for the purpose of ensemble modeling. Ensemble models combining preOp_ML with DL-IVSS_only, DL-IVSS_PCFs 3, DL-IVSS_PCFs 11, and DL-IVSS_PCVSFs 28 were labeled as Ensemble_only, Ensemble_PCFs 3, Ensemble_PCFs 11, and Ensemble_PCVSFs 28, respectively. For numerical features, robust regularization techniques were applied for stable learning of the preoperative PO-AKI risk prediction model.

### Deep-learning algorithm development using time-series intraoperative vital sign records

**Fig 1:**
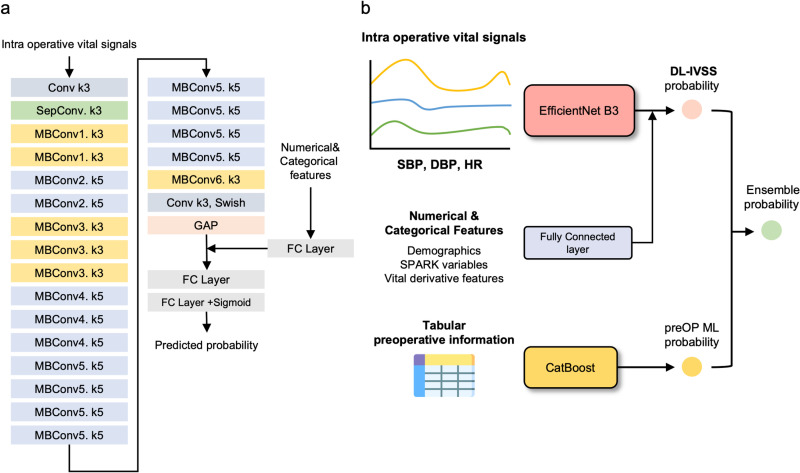
Study design and deep-learning model architecture. a. Design of the deep-learning model to construct the prediction models. b. Overall study design for prediction of postoperative AKI or Critical AKI. Conv, Convolutional layer; SepConv, Separable convolutional layer; MBConv, Mobile inverted bottleneck convolutional layer (numbers after MBConv indicate layer depth); k3/k5, kernel size 3 or 5; GAP, Global average pooling; FC, Fully connected layer; Swish, Swish activation function; DBP, Diastolic blood pressure, SBP, Systolic blood pressure; HR, Heart rate; DL-IVSS, A deep-learning algorithm leveraging time-series intraoperative vital sign signals; preOp ML, A machine learning model with 103 baseline characteristics.

We utilized a CNN-based model to develop DL-IVSS, leveraging its ability to automatically learn complex patterns and to extract hidden characteristics from input data. Among several architectures tested–ResNet34, ResNet50, EfficientNet b3, EfficientNet b5 and a Long short-term memory (LSTM) model–EfficientNet b3 demonstrated the best prediction power, efficient structure, achieving high performance with a relatively low number of parameters (S3 Table, Fig 1A). We incorporated tabular data into the DL-IVSS by concatenating it with the signal-embedded vector from the convolutional layers, processing real-time intraoperative bio-signals. The final prediction was made using fully connected layers. In the ensemble model, we integrated CNN with a Catboost model, a machine-learning-based method proficient in handling tabular information (Fig 1B).

### Visualizing feature attributions on deep-learning algorithms

We employed an explainable AI method to elucidate causal inference and interpret the model’s predictions. The Integrated Gradients (IG) method was used, as it is known for its capability to handle multi-modal data effectively [18]. IG quantifies feature importance by calculating the gradients of the model’s output with respect to the input features.

To visualize feature attributions, we focused on the absolute magnitude of IG values to assess the relative importance of different modalities, such as intraoperative vital sign signals and preoperative tabular information, as well as individual features within the tabular data.

### Statistical analysis

For performance evaluation, area under the receiver operating characteristic curve (AUROC), positive predictive value (PPV), negative predictive value (NPV), sensitivity, and specificity were calculated in this study. AUROC was used to evaluate and compare the discriminative performancesof the developed deep-learning models. The 95% CI of AUROC was obtained through the DeLong Test [19], and two-sided p-value of < 0.05 was considered statistically significant for rejecting the null hypothesis. Sensitivity/specificity-related indices were used to evaluate whether a pre-determined sensitivity/specificity threshold may provide tolerable detection of those at risk of PO-AKI and we set the 95% threshold value for the indices. This approach was used to suggest a binary threshold may be a useful way to easily alarm the attending physicians about the potential risk of PO-AKI from a practical perspective . All statistical analyses were performed with R (version 4.2.2) and Python (version 3.6.2).

## Results

### Study population

**Fig 2:**
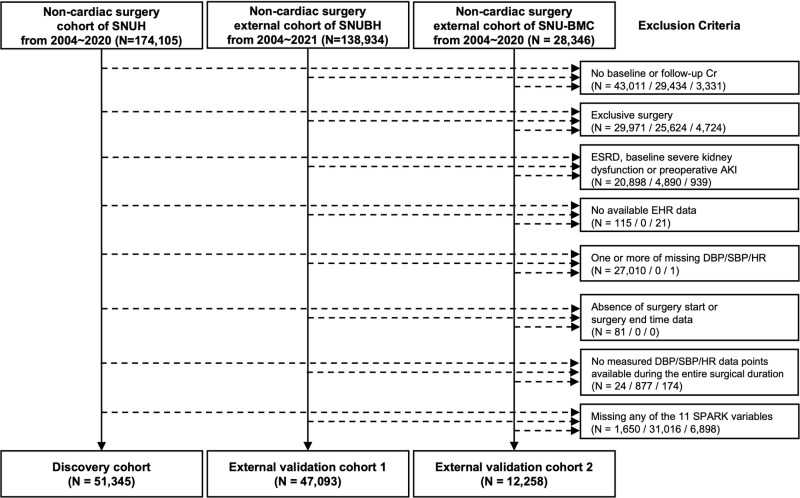
Study flow diagram. Flow chart of dataset construction. SNUH, Seoul National University Hospital; SNUBH, Seoul National University Bundang Hospital; SNU-BMC, Seoul National University Boramae Medical Center; Cr, Creatinine; ESRD, End stage renal disease; AKI, Acute kidney injury; EHR, Electronic Health Records; DBP, Diastolic blood pressure; SBP, Systolic blood pressure; HR, Heart rate.

We screened a total of 174,105 patients from the developmental cohort, 138,934 from EVC 1, and 28,346 from EVC 2 undergoing non-cardiac major surgeries. After applying exclusion criteria, the final 51,345 patients were included in the developmental cohort. For the external validation cohorts, 47,093 patients from EVC 1 and 12,258 patients from EVC 2 with complete intraoperative vital sign signals and related clinicodemographic information available were included for assessment for PO-AKI event occurrence (Fig 2).

### Preoperative baseline characteristics

The baseline characteristics of the three study hospitals, as shown in Table 1, reveal nuanced demographic and clinical profiles. The proportions of patients with a baseline eGFR <  60 mL/min/1.73 m^2^ ranged from 5.6% to 7.8% across the institutions. Patients in the developmental cohort were predominantly male and underwent more emergent operations. However, they had lower rates of diabetes, anemia, and preoperative RAAS blockade use compared to the two validation cohorts. Conversely, patients in the EVCs were more hyponatremic, although their median expected operation durations were shorter than the other two cohorts. PO-AKI occurred in 3,188 (6.2%) cases in the developmental cohort, 2,519 (5.3%), and 579 (4.7%) in the EVCs, whereas critical AKI occurred similarly across the three cohorts. The baseline clinical characteristics were generally worse in patients with PO-AKI in each study hospital than in those without AKI (S4 Table). Namely, PO-AKI patients had older age, lower eGFR, more frequent dipstick albuminuria, longer surgery duration, higher prevalence of diabetes and RAAS blockade use and abnormal laboratory profiles.

**Table 1:** Baseline characteristics.

Preoperative risk factors	Developmental cohort (N = 51,345)	EVC 1 (N = 47,093)	EVC 2 (N = 12,258)
Age (years)	60.0 [49.0,69.0]	61.0 [50.0,70.0]	66.0 [55.0,73.0]
<40	6,919 (13.5)	5,181 (11.0)	1,126 (9.2)
≥40 and < 60	18,472 (36.0)	16,100 (34.2)	3,080 (25.1)
≥60 and < 80	24,132 (47.0)	23,614 (50.1)	7,162 (58.4)
≥80	1822 (3.5)	2,198 (4.7)	890 (7.3)
eGFR (mL/min per 1.73 m^2^)	89.9 [76.0,102.0]	93.2 [82.2,103.3]	91.5 [80.3,101.3]
≥60	47,743 (93.0)	44,463 (94.4)	11,305 (92.2)
≥45 and < 60	2,775 (5.4)	1884 (4.0)	648 (5.3)
≥30 and < 45	639 (1.2)	565 (1.2)	236 (1.9)
≥15 and < 30	188 (0.4)	181 (0.4)	69 (0.6)
Dipstick albuminuria (albumin ≥ 1+)	5,738 (11.2)	3,229 (6.9)	1,343 (11.0)
Sex			
Female	23,137 (45.1)	23,167 (49.2)	7,028 (57.3)
Male	28,208 (54.9)	23,926 (50.8)	5,230 (42.7)
Expected surgical duration (hours)	3.0 [2.0,4.0]	3.0 [2.0,4.0]	2.0 [2.0,3.0]
Emergency operation	3,772 (7.3)	702 (1.5)	230 (1.9)
Diabetes mellitus	6,332 (12.3)	7,766 (16.5)	2,431 (19.8)
RAAS blockade use	3,372 (6.6)	1969 (4.2)	1,791 (14.6)
Hypoalbuminemia (<3.5 g/dL)	5,375 (10.5)	11,689 (24.8)	2,385 (19.5)
Anemia (<12 g/dL for female, 13 g/dL for male)	15,116 (29.4)	18,660 (39.6)	5,695 (46.5)
Hyponatremia (<135 mEq/L)	1,491 (2.9)	1,045 (2.2)	708 (5.8)

Representative characteristics included in the DL-IVSS modeling are presented. Data are presented as median [interquartile range] for continuous variables and n (%) for categorical variables.

EVC, External validation cohort; eGFR, Estimated glomerular filtration rate; RAAS, Renin-angiotensin-aldosterone system

### Intraoperative vital sign signals

We analyzed intraoperative vital sign signals such as diastolic and systolic BP as well as heart rate parameters, including approximately 9,300,000 signal points from the developmental cohort, approximately 6,300,000 from EVC 1, and ~ 1,600,000 from EVC 2, after excluding artifacts and outliers (S1–S3 Figs). To summarize the collected intraoperative vital sign data, we provided key metrics, including the total length of the data points, time with mean BP <  65 mmHg, time with heart rate <  60 or >  100, as well as indices for mean, change, and variation, as shown in S2 Table.

### PO-AKI prediction model development based on deep-learning algorithms

We first constructed the DV-IVSS_only and evaluated whether inclusion of multi-level data improved predictive performance. For comparison, we used the clinical SPARK model as the reference without incorporating deep-learning techniques. Notably, the SPARK model consistently demonstrated acceptable discriminative abilities, with AUROC values exceeding the threshold of 0.7 for both PO-AKI and critical AKI risks (Table 2).

**Table 2:** Discriminative performances for postoperative AKI risk by intraoperative vital sign signals.

Outcome	Hospital (Cases N (%))	Method	AUROC	p-value (vs. SPARK)	p-value (vs. preceding model)	Balanced Accuracy	NPV (Spec 0.95)	PPV (Sens 0.95)
PO-AKI	Developmental cohort (3,188 (6.2%))	SPARK	0.724 (0.707, 0.745)	–		0.664 (0.644, 0.685)	0.951 (0.945, 0.957)	0.072 (0.063, 0.079)
		DL-IVSS_only	0.707 (0.687, 0.723)	0.165		0.666 (0.646, 0.687)	0.954 (0.948, 0.960)	0.066 (0.059, 0.073)
		DL-IVSS_PCFs 3	0.748 (0.733, 0.761)	0.009	<0.001	0.691 (0.672, 0.712)	0.954 (0.948, 0.960)	0.069 (0.062, 0.076)
		DL-IVSS_PCFs 11	0.765 (0.745, 0.778)	<0.001	0.016	0.708 (0.690, 0.729)	0.955 (0.949, 0.960)	0.073 (0.065, 0.081)
	EVC 1 (2,519 (5.3%))	SPARK	0.697 (0.689, 0.708)	–		0.646 (0.637, 0.655)	0.955 (0.953, 0.957)	0.062 (0.060, 0.065)
		DL-IVSS_only	0.637 (0.623, 0.647)	<0.001		0.601 (0.591, 0.611)	0.950 (0.948, 0.952)	0.061 (0.059, 0.064)
		DL-IVSS_PCFs 3	0.721 (0.713, 0.729)	<0.001	<0.001	0.662 (0.653, 0.671)	0.954 (0.952, 0.956)	0.060 (0.058, 0.062)
		DL-IVSS_PCFs 11	0.716 (0.706, 0.722)	<0.001	0.220	0.655 (0.644, 0.665)	0.956 (0.954, 0.958)	0.061 (0.059, 0.064)
	EVC 2 (579 (4.7%))	SPARK	0.745 (0.728, 0.759)	–		0.689 (0.669, 0.708)	0.963 (0.959, 0.966)	0.060 (0.055, 0.065)
		DL-IVSS_only	0.607 (0.583, 0.631)	<0.001		0.588 (0.568, 0.608)	0.955 (0.951, 0.958)	0.050 (0.046, 0.054)
		DL-IVSS_PCFs 3	0.728 (0.708, 0.747)	0.051	<0.001	0.682 (0.664, 0.704)	0.960 (0.956, 0.963)	0.051 (0.047, 0.056)
		DL-IVSS_PCFs 11	0.761 (0.733, 0.782)	<0.001	<0.001	0.701 (0.682, 0.720)	0.962 (0.959, 0.966)	0.054 (0.050, 0.059)
Critical AKI	Developmental cohort (529 (1.0%))	SPARK	0.796 (0.756, 0.841)	–		0.729 (0.684, 0.776)	0.992 (0.989, 0.994)	0.014 (0.011, 0.018)
		DL-IVSS_only	0.724 (0.666, 0.790)	0.015		0.686 (0.641, 0.731)	0.992 (0.989, 0.994)	0.011 (0.008, 0.015)
		DL-IVSS_PCFs 3	0.790 (0.750, 0.826)	0.770	0.003	0.727 (0.679, 0.770)	0.993 (0.991, 0.996)	0.014 (0.010, 0.018)
		DL-IVSS_PCFs 11	0.816 (0.787, 0.861)	0.27	0.182	0.755 (0.715, 0.793)	0.993 (0.991, 0.996)	0.013 (0.010, 0.017)
	EVC 1 (448 (1%))	SPARK	0.727 (0.712, 0.746)	–	–	0.664 (0.642, 0.684)	0.993 (0.992, 0.994)	0.013 (0.011, 0.014)
		DL-IVSS_only	0.729 (0.706, 0.749)	0.905	–	0.671 (0.649, 0.692)	0.992 (0.991, 0.993)	0.012 (0.011, 0.014)
		DL-IVSS_PCFs 3	0.755 (0.742, 0.765)	0.019	0.039	0.695 (0.673, 0.718)	0.992 (0.991, 0.993)	0.013 (0.012, 0.015)
		DL-IVSS_PCFs 11	0.740 (0.719, 0.760)	0.123	0.200	0.677 (0.654, 0.699)	0.993 (0.992, 0.993)	0.012 (0.011, 0.013)
	EVC 2 (111 (0.9%))	SPARK	0.756 (0.715, 0.797)	–	–	0.715 (0.668, 0.758)	0.993 (0.992, 0.995)	0.013 (0.010, 0.015)
		DL-IVSS_only	0.716 (0.672, 0.781)	0.207	–	0.686 (0.644, 0.730)	0.992 (0.990, 0.994)	0.012 (0.009, 0.014)
		DL-IVSS_PCFs 3	0.750 (0.706, 0.788)	0.813	0.212	0.709 (0.669, 0.748)	0.992 (0.991, 0.994)	0.013 (0.010, 0.015)
		DL-IVSS_PCFs 11	0.794 (0.750, 0.818)	0.007	0.045	0.747 (0.705, 0.787)	0.993 (0.992, 0.994)	0.012 (0.010, 0.015)

Performance metrics are presented as the calculated values with 95% confidence intervals in parentheses. The “p-value (vs. preceding model)” column represents the p-value comparing the performance of each deep-learning model with the immediately preceding model in the table. For example, the p-value in the DL-IVSS_PCFs 11 row compares its performance with DL-IVSS_PCFs 3. The “NPV (Spec 0.95)” column represents NPV when a specificity threshold of 95% was applied. The “PPV (Sens 0.95)” column represents PPV when a sensitivity threshold of 95% was applied.

PO-AKI, Postoperative acute kidney injury; EVC, External validation cohort; AUROC, Area under the receiver operating characteristic curve; PPV, Positive predictive value; NPV, Negative predictive value; DL-IVSS_only, A deep-learning algorithm leveraging only time-series intraoperative vital sign signals; DL-IVSS_PCFs 3, A deep-learning algorithm leveraging time-series intraoperative vital sign signals and preoperative clinical features 3; DL-IVSS_PCFs 11, A deep-learning algorithm leveraging time-series intraoperative vital sign signals and preoperative clinical features 11

Then, we scrutinized the DL-IVSS algorithm performance (DL-IVSS_only), which demonstrated acceptable discriminative performance with an AUROC of 0.707 (95% CI 0.684, 0.730) in the developmental cohort, although falling below 0.70 in the EVCs for PO-AKI prediction. However, the DL-IVSS indicated promising predictive capabilities with AUROC values above 0.7 in both developmental and validation cohorts for predicting critical AKI.

We further enhanced the DL-IVSS model by incorporating three basic clinical variables: age, sex, and eGFR (DL-IVSS_PCFs 3), which significantly improved their discriminative power for both PO-AKI and critical AKI. Notably, even with the addition of a limited number of clinical variables (DL-IVSS_PCFs 11), the DL-IVSS models outperformed the SPARK model in both the developmental cohort and EVC 1 for predicting PO-AKI and critical AKI. Moreover, integrating the preoperative SPARK features into the DL-IVSS model (DL-IVSS_PCVSFs 11) demonstrated further improvement in discriminative performance. In contrast, the addition of intraoperative vital sign summary data did not significantly improve predictive accuracy (Tables 2 and S5).

### Feature importance of the DL-IVSS algorithm

**Fig 3:**
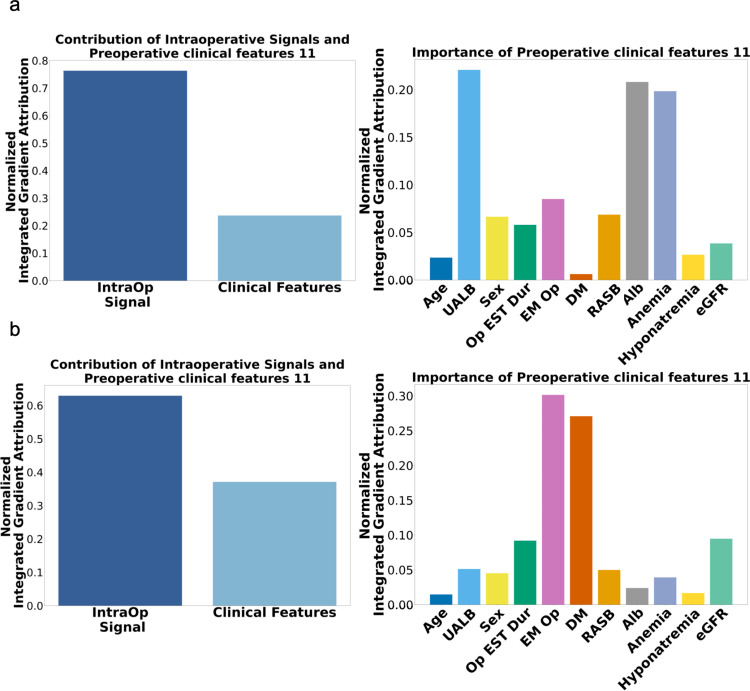
Feature importance of the deep-learning-based models using the intraoperative vital sign signals. **a.** Results towards PO-AKI outcome. **b.** Results towards critical AKI outcome. IntraOp Signal, Intraoperative vital sign signal; UALB, Dipstick urine albumin; Op EST Dur, estimated surgery time; EM Op, emergent operation; DM, diabetes mellitus; RASB, Preoperative use of renin-angiotensin-aldosterone inhibitors; Alb, serum albumin; eGFR, Estimated glomerular filtration rate.

During the model development process, we analyzed the relative contribution of input data to risk prediction and found that intraoperative vital sign signals had the greatest impact on predicting PO-AKI, surpassing other preoperative risk factors and vital sign summary information (Fig 3A). This pattern remained consistent when predicting critical AKI (Fig 3B). These findings underscore the pivotal role of intraoperative physiological parameters in AKI risk prediction, as they accounted for most of the predictive performance demonstrated by the deep-learning models.

### Integrative ensemble model incorporating the DL-IVSS and preoperative machine-learning model

Next, we compared the ensemble model performances including Ensemble_only consisted of preoperative ML with DL-IVSS (preoperative ML model results are available in S6 Table), Ensemble_PCFs 3, Ensemble_PCFs 11, and Ensemble_PCVSFs 28 with DL-IVSS_PCFs 11 model performance. The ensemble models either outperformed or were at least not inferior for predicting both PO-AKI (AUROC, 0.790, 0.796) and critical AKI (AUROC, 0.845, 0.859) in the developmental cohort as well as in two EVCs (Tables 3 and S7). Among the ensemble models, Ensemble_PCFs 11 showed the best performance in the overall cohorts for predicting both PO-AKI and critical AKI. Given the lack of superior performance and the potential for overfitting in the 28-feature model, we excluded it from further consideration. Instead, we retained the Ensemble_PCFs 11 model as the main model for subsequent analyses.

**Table 3:** Discriminative performances for postoperative AKI risk by ensemble models.

Outcome	Hospital	Method	AUROC	p-value (vs. DL-IVSS_PCFs 11)	p-value (vs. preceding model)	Balanced Accuracy	NPV (Spec 0.95)	PPV (Sens 0.95)
PO-AKI	Developmental cohort (3,188 (6.2%))	DL-IVSS_PCFs 11	0.765 (0.745, 0.778)	–		0.708 (0.690, 0.729)	0.955 (0.949, 0.960)	0.073 (0.065, 0.081)
		Ensemble_only	0.792 (0.774, 0.807)	<0.001		0.732 (0.713, 0.750)	0.958 (0.952, 0.964)	0.077 (0.068, 0.086)
		Ensemble_PCFs 3	0.790 (0.772, 0.817)	<0.001	0.533	0.724 (0.706, 0.745)	0.960 (0.954, 0.965)	0.078 (0.069, 0.086)
		Ensemble_PCFs 11	0.795 (0.784, 0.807)	<0.001	0.112	0.732 (0.714, 0.752)	0.958 (0.953, 0.964)	0.076 (0.068, 0.084)
	EVC 1 (2,519 (5.3%))	DL-IVSS_PCFs 11	0.716 (0.706, 0.722)	–		0.655 (0.644, 0.665)	0.956 (0.954, 0.958)	0.061 (0.059, 0.064)
		Ensemble_only	0.759 (0.750, 0.768)	<0.001		0.699 (0.689, 0.707)	0.961 (0.959, 0.963)	0.067 (0.065, 0.070)
		Ensemble_PCFs 3	0.764 (0.754, 0.773)	<0.001	<0.001	0.700 (0.692, 0.710)	0.961 (0.959, 0.962)	0.066 (0.063, 0.068)
		Ensemble_PCFs 11	0.762 (0.755, 0.767)	<0.001	0.094	0.696 (0.687, 0.704)	0.962 (0.960, 0.964)	0.062 (0.060, 0.064)
	EVC 2 (579 (4.7%))	DL-IVSS_PCFs 11	0.761 (0.733, 0.782)	–		0.701 (0.682,0.720)	0.962 (0.959, 0.966)	0.054 (0.050, 0.059)
		Ensemble_only	0.766 (0.742, 0.779)	0.583		0.705 (0.685, 0.722)	0.964 (0.961, 0.967)	0.057 (0.052, 0.061)
		Ensemble_PCFs 3	0.776 (0.752, 0.801)	0.030	0.014	0.707 (0.687, 0.726)	0.965 (0.962, 0.968)	0.060 (0.055, 0.064)
		Ensemble_PCFs 11	0.786 (0.770, 0.802)	<0.001	0.001	0.715 (0.697, 0.733)	0.967 (0.964, 0.970)	0.056 (0.052, 0.060)
Critical AKI	Developmental cohort (529 (1.0%))	DL-IVSS_PCFs 11	0.816 (0.787,0.861)	–		0.755 (0.715,0.793)	0.993 (0.991, 0.996)	0.013 (0.010,0.017)
		Ensemble_only	0.846 (0.817, 0.880)	0.027		0.773 (0.725, 0.815)	0.994 (0.991, 0.996)	0.017 (0.012,0.022)
		Ensemble_PCFs 3	0.859 (0.825, 0.868)	<0.001	0.170	0.785 (0.737, 0.824)	0.994 (0.991, 0.996)	0.018 (0.013, 0.024)
		Ensemble_PCFs 11	0.849 (0.812, 0.898)	<0.001	0.246	0.778 (0.736, 0.820)	0.993 (0.991, 0.996)	0.016 (0.011, 0.020)
	EVC 1 (448 (1%))	DL-IVSS_PCFs 11	0.740 (0.719, 0.760)	–		0.677 (0.654, 0.699)	0.993 (0.992, 0.993)	0.012 (0.011, 0.013)
		Ensemble_only	0.821 (0.797, 0.829)	<0.001		0.754 (0.731, 0.772)	0.995 (0.994, 0.995)	0.014 (0.012, 0.015)
		Ensemble_PCFs 3	0.828 (0.812, 0.845)	<0.001	0.038	0.758 (0.736, 0.781)	0.994 (0.994, 0.995)	0.016 (0.014, 0.017)
		Ensemble_PCFs 11	0.811 (0.801, 0.826)	<0.001	<0.001	0.740 (0.719, 0.762)	0.994 (0.993, 0.995)	0.014 (0.012, 0.015)
	EVC 2 (111 (0.9%))	DL-IVSS_PCFs 11	0.794 (0.750, 0.818)	–		0.747 (0.705, 0.787)	0.993 (0.992, 0.994)	0.012 (0.010, 0.015)
		Ensemble_only	0.817 (0.777, 0.848)	0.290		0.757 (0.718, 0.796)	0.994 (0.992, 0.995)	0.015 (0.013, 0.018)
		Ensemble_PCFs 3	0.820 (0.797, 0.859)	0.167	0.590	0.760 (0.722, 0.793)	0.994 (0.992, 0.995)	0.017 (0.014, 0.021)
		Ensemble_PCFs 11	0.832 (0.807, 0.857)	0.011	0.074	0.757 (0.722, 0.792)	0.994 (0.993, 0.995)	0.014 (0.012, 0.017)

Performance metrics are presented as the calculated values with 95% confidence intervals in parentheses. The “p-value (vs. preceding model)” column represents the p-value comparing the performance of each deep-learning model with the immediately preceding model in the table. For example, the p-value in the Ensemble_PCFs 11 row compares its performance with Ensemble_PCFs 3. The “NPV (Spec 0.95)” column represents NPV when a specificity threshold of 95% was applied. The “PPV (Sens 0.95)” column represents PPV when a sensitivity threshold of 95% was applied.

PO-AKI, Postoperative acute kidney injury; EVC, External validation cohort; AUROC, Area under the receiver operating characteristic curve; PPV, Positive predictive value; NPV, Negative predictive value; DL-IVSS_PCFs 11, A deep-learning algorithm leveraging time-series intraoperative vital sign signals and preoperative clinical features 11; Ensemble_only, An ensemble model combining preOp_ML and DL-IVSS_only; Ensemble_PCFs 3, An ensemble model combining preOp_ML and DL-IVSS_PCFs 3; Ensemble_PCFs 11, An ensemble model combining preOp_ML and DL-IVSS_PCFs 11

### Calibration plot

**Fig 4:**
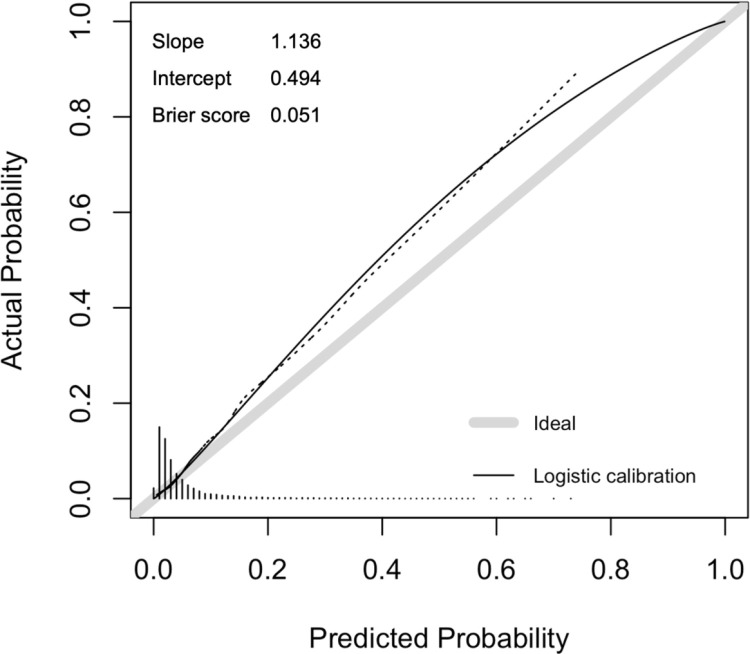
Calibration plot for the Ensemble_PCFs 11. The solid black line represents the logistic calibration curve, while the dashed line shows the nonparametric calibration curve that directly connects observed data points, with the grey line showing ideal calibration. The Brier score indicates the mean squared difference between predicted and actual probabilities, where values closer to 0 represent better calibration. The histogram shows the distribution of predicted probabilities.

We performed a calibration analysis for Ensemble_PCFs 11 to evaluate the agreement between observed and predicted probabilities, with results summarized in Fig 4. The calibration slope was 1.136, close to the ideal value of 1, indicating that the model’s predicted probabilities scale well with observed outcomes. The calibration intercept was 0.494, suggesting a slight underestimation of risk across all probability ranges, as predicted probabilities were systematically lower than observed probabilities.

The Brier score, which quantifies the mean squared difference between predicted probabilities and actual outcomes (where 0 is optimal), was 0.051, reflecting strong model reliability and minimal prediction errors. The calibration curve closely aligns with the ideal diagonal line with minor deviations in the lower probability range (0.1–0.3), where the model slightly underestimates risk. The histogram revealed that a clustering of predicted probabilities in the lower range, a common occurrence in imbalanced datasets. Additionally, logistic calibration line demonstrates slight deviations at lower probabilities, further supporting the observation of minor underestimation.

### Practical application scenario

**Fig 5:**
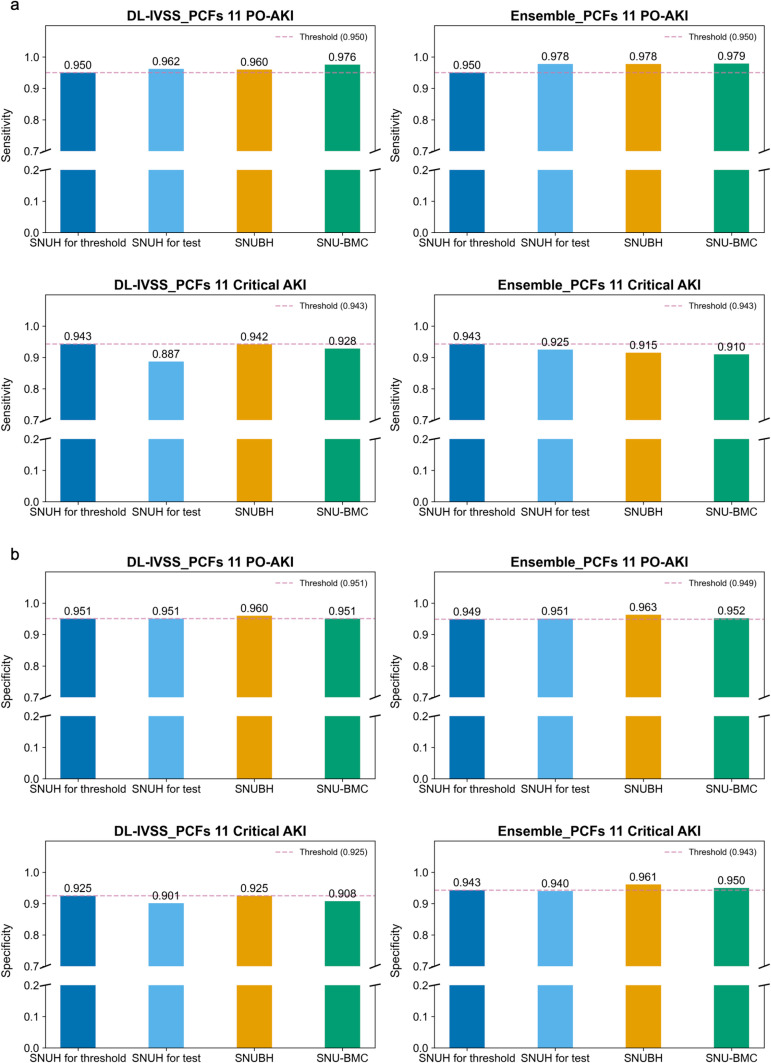
Discriminative performances for postoperative AKI and critical AKI risk by intraoperative vital sign signals at preset thresholds. **a.** Model performance in each study data when sensitivity 95% threshold was applied. **b.** Model performance in each study data when specificity 95% threshold was applied. DL-IVSS_PCFs 11, A deep-learning algorithm leveraging time-series intraoperative vital sign signals and preoperative clinical features 11; Ensemble_PCFs 11, A ensemble model combining preOp_ML and DL-IVSS_PCFs 11; PO-AKI, Postoperative acute kidney injury; SNUH, Seoul National University Hospital; SNUBH, Seoul National University Bundang Hospital; SNU-BMC, Seoul National University Boramae Medical Center.

To suggest a practical binary stratification of PO-AKI risk, we set the 95% sensitivity and specificity threshold using the development data. Then, we applied the threshold to the study data to validate their performances. Based on the above results, we used the deep-learning model incorporating incorporating both the eleven SPARK features and intraoperative vital sign signals as it showed the best discriminative performance for the outcomes (Fig 5 and S8 and S9 Tables). When the sensitivity threshold was applied, the model detected 96.2% of PO-AKI events and 88.7% of critical AKI cases in the internal test data. In the two EVCs, the detection rate for PO-AKI and critical AKI were 96.0/94.2% and 97.6/92.8%, respectively. Using the specific threshold, the specificity rates in the internal test set were 95.1% for PO-AKI and 90.1% for critical AKI, respectively. Similarly, the specificity results in the EVCs exceeded >  95% for both outcomes, demonstrating the robustness and practical utility of the model thresholds.

## Discussion

In this study, we incorporated complex, large-scale intraoperative vital sign signals at a minute-level resolution into a PO-AKI risk prediction model. The DL-IVSS showed comparable performance to a conventional AKI risk prediction model, even without demographic or laboratory values. When basic clinical variables were added, the DL-IVSS outperformed the conventional model in predicting PO-AKI and critical AKI events, reflecting its clinical relevance. Finally, we developed and validated an ensembled deep-learning model combining preoperative and intraoperative vital signs, which achieved the highest predictive accuracy.

Predicting of PO-AKI in non-cardiac major surgery remains challenging due to its lower incidence compared to cardiac operations [3] but it is essential because of its significant impact on patient prognosis. Recently, machine-learning-based algorithms have been introduced alongside conventional PO-AKI risk models [7,14,20]. However, deep-learning models handling complex vital sign signals themselves including millions of data points from a large-scale cohort are rare, although PO-AKI risk is tightly associated with BP or heart rate alteration during surgery [5]. Previous studies often relied on summary statistics (mean, minimum or maximum values, and measurements for variations) for intraoperative vital signs rather than using complete real-world data. Our study uniquely incorporated full intraoperative data for systolic and diastolic BP and heart rate in a deep-learning model, demonstrating superior performance to conventional PO-AKI risk prediction model. In addition, we established practical thresholds for highly sensitive and specific PO-AKI risk prediction, providing actionable cut-off values. Supported by large-scale external validation cohorts and the inclusion of critical AKI outcomes, our findings highlight the potential of deep-learning based model incorporating real-time vital sign signals with robust clinical utility, expanding the application to non-cardiac surgeries [20].

Intraoperative blood pressure is critically associated with PO-AKI risk [5]. This is due to its impact on kidney blood flow which is linked to ischemic injury. Both BP reduction and variability can cause short-term maladaptation. Heart rate is another important vital sign significantly associated with AKI risk. In our DL-IVSS, intraoperative vital signs were more influential than preoperative baseline characteristics in predicting PO-AKI risk, as shown by “feature importance” analysis. Considering that our models predicted PO-AKI risk comparable to a conventional PO-AKI risk prediction model even without basic clinical information (e.g., age, sex, or baseline kidney function), the importance of intraoperative vital signs in regard to PO-AKI risk is underscored. On the other hand, the addition of summary-level vital sign data did not improve the model performances when compared to the model including IVSS. Thus, DL-IVSS may be a valid method to reflect both short-term and overall alterations in BP and heart rate in relation to the risk of PO-AKI.

We used the CNN-based EfficientNet B3 to extract significant features from intraoperative vital signs. CNNs are well-established for processing biosignals with their capacity to extract hierarchical features, which is particularly advantageous for accurate AKI prediction. This allows the model to analyze both short-term fluctuations and long-term trends. While most studies on AKI prediction have mainly used Recurrent Neural Networks-based models [21,22], CNNs offer advantages such as reduced susceptibility to the vanishing gradient problem and greater computational efficiency [23]. In addition, we employed an ensemble technique by combining the CNN with the CatBoost model, which excels at extracting information from structured data like demographic variables. This hybrid approach further enhanced the performance of our prediction model by incorporating additional information that cannot be captured from intraoperative vital signs alone. This multi-faceted approach leverages the strengths of both CNN and CatBoost, resulting in improved predictive accuracy for PO-AKI.

The current model can predict PO-AKI and critical AKI risk immediately after surgery by integrating preoperative information and intraoperative vital signs. Using the sensitive and specific thresholds identified in this study, the model can support clinical decisions, such as determining the need for follow-up kidney function assessments, a nephrologist referral, or routine postoperative care. The high NPV values of the model underscore its reliability in ruling out patients who are unlikely to develop PO-AKI, allowing clinicians to focus resources on high-risk individuals. The low PPV of our model is a result of the overall low event rate of PO-AKI. The model’s clinical utility lies in its ability to identify patients who may not exhibit immediate symptoms but are at potential risk, emphasizing its role in proactive and preventive care strategies. Additionally, this study highlights a future direction for PO-AKI risk prediction: the potential for real-time intraoperative vital sign monitoring with continuous updates for PO-AKI risk assessment during surgery. A system based on the DL-IVSS framework could provide real-time alerts to attending clinicians, enabling timely interventions (e.g., BP stabilization) to mitigate the risk. Further research is warranted to explore the benefits of real-time vital sign monitoring and stabilization as well as its impact on PO-AKI risk reduction. This could be achieved through additional deep-learning studies focusing on real-time monitoring and intervention strategies.

Our study has several limitations. First, as the field of deep learning is is a rapidly evolving field, further comparison of the implemented deep-learning methods would be needed to ensure the validity of the constructed model. Still, additional models may have superior performance, thus, the constructed model should be updated following technology innovations. Second, the study was a single-nation study, thus, although the study was powered by external hospital validation, additional validation in a different nation or society is necessary. Third, some blank of the intraoperative vital sign data was imputed by zero padding. A thorough collection of minute-scale intraoperative vital information, also including other types of available data (e.g., blood oxygen saturation), minimizing missing values may further improve the model performance. Lastly, the current analysis is based on retrospectively collected routine data. Prospective validation of this model may confirm its clinical utility, and further expansion could be explored by incorporating novel biomarkers associated with AKI.

In conclusion, our study demonstrates that the DL-IVSS model, incorporating large-scale intraoperative vital signs, is a valid and effective tool for predicting PO-AKI risk following non-cardiac surgeries. The model’s performance was further enhanced with the addition of preoperative clinical variables. Future deep-learning research should prioritize the inclusion of intraoperative vital sign data to develop robust, real-time prediction models for PO-AKI risk. With continued advancements, deep-learning strategies may facilitate early detection and management of PO-AKI, ultimately improving patient outcomes.

## Supporting information

S1 Checklist Transparent reporting of a multivariable prediction model for individual prognosis or diagnosis with artificial intelligence (TRIPOD-AI) checklist. (DOCX)

S1 Table Missing count and missing rate of preoperative clinical variables. (DOCX)

S2 Table Characteristics of the intraoperative vital sign signal derived variables. (DOCX)

S3 Table Performance of different model architectures for postoperative AKI risk prediction. (DOCX)

S4 Table Characteristics of the study variables according to the presence of postoperative AKI in each study hospital. (DOCX)

S5 Table Discriminative performances for postoperative AKI risk by intraoperative vital sign signals and additional summary-level vital sign information. (DOCX)

S6 Table Discriminative performances for postoperative AKI risk by preoperative ML model. (DOCX)

S7 Table Discriminative performances for postoperative AKI risk by ensemble models and additional summary-level vital sign information. (DOCX)

S8 Table Model performance in each study data when sensitivity 95% threshold was applied. (DOCX)

S9 Table Model performance in each study data when specificity 95% threshold was applied. (DOCX)

S1 Fig Detailed data preprocessing and dataset construction flow for the developmental cohort (SNUH). SNUH = Seoul National University Hospital; EHR = Electronic Health Records; DBP = Diastolic blood pressure; SBP = Systolic blood pressure; IDBP = Invasive diastolic blood pressure; ISBP = Invasive systolic blood pressure; HR = Heart rate.
(DOCX)

S2 Fig Detailed data preprocessing and dataset construction flow for the external validation cohort 1 (SNUBH). SNUBH = Seoul National University Bundang Hospital; EHR = Electronic Health Records; DBP = Diastolic blood pressure; SBP = Systolic blood pressure; IDBP = Invasive diastolic blood pressure; ISBP = Invasive systolic blood pressure; HR = Heart rate.
(DOCX)

S3 Fig Detailed data preprocessing and dataset construction flow for the external validation cohort 2 (SNU-BMC). SNU-BMC = Seoul National University Boramae Medical Center; EHR = Electronic Health Records; DBP = Diastolic blood pressure; SBP = Systolic blood pressure; IDBP = Invasive diastolic blood pressure; ISBP = Invasive systolic blood pressure; HR = Heart rate.
(DOCX)
