# Supplementary material for: A deep-learning algorithm using real-time collected intraoperative vital sign signals for predicting acute kidney injury after major non-cardiac surgeries: A modelling study
Source: PLoS Med. 2025 Apr 29;22(4):e1004566. doi: 10.1371/journal.pmed.1004566 (PMC12040160; doi:10.1371/journal.pmed.1004566)
Supplement: S1 Table — (DOCX) [file pmed.1004566.s002.docx]

**S1 Table. Missing count and missing rate of preoperative clinical variables.**

| Preoperative clinical variables | Development cohort | | EVC1 | | EVC2 | |
| --- | --- | --- | --- | --- | --- | --- |
|  | missing count | missing rate (%) | missing count | missing rate (%) | missing count | missing rate (%) |
| Sex | 0 | 0 | 0 | 0 | 0 | 0 |
| Current-smoker | 14752 | 28.731 | 204 | 0.433 | 21 | 0.171 |
| Ex-smoker | 209 | 0.407 | 125 | 0.265 | 21 | 0.171 |
| Operation department | 0 | 0 | 0 | 0 | 0 | 0 |
| Type of admission | 0 | 0 | 0 | 0 | 48 | 0.392 |
| Operation type | 0 | 0 | 0 | 0 | 0 | 0 |
| Operation anesthesia | 208 | 0.405 | 0 | 0 | 133 | 1.085 |
| ASA grade | 805 | 1.568 | 1275 | 2.707 | 339 | 2.765 |
| NYHA grade | 27518 | 53.594 | 47093 | 100 | 12221 | 99.698 |
| Presence of patient-described history of diabetes mellitus | 0 | 0 | 1273 | 2.703 | 0 | 0 |
| Hypertension | 0 | 0 | 1273 | 2.703 | 0 | 0 |
| Kidney disease | 0 | 0 | 47093 | 100 | 0 | 0 |
| Heart disease | 0 | 0 | 1273 | 2.703 | 0 | 0 |
| Liver disease | 0 | 0 | 47093 | 100 | 0 | 0 |
| Tuberculosis | 0 | 0 | 47093 | 100 | 0 | 0 |
| Thyroid disease | 0 | 0 | 47093 | 100 | 0 | 0 |
| Asthma | 0 | 0 | 47093 | 100 | 0 | 0 |
| COPD | 0 | 0 | 47093 | 100 | 0 | 0 |
| Hematologic disease | 0 | 0 | 47093 | 100 | 0 | 0 |
| Neurologic disease | 0 | 0 | 47093 | 100 | 0 | 0 |
| Other organ comorbidity | 0 | 0 | 47093 | 100 | 0 | 0 |
| Pregnancy history | 0 | 0 | 47093 | 100 | 0 | 0 |
| Vascular disease | 0 | 0 | 47093 | 100 | 0 | 0 |
| Diagnosis record of diabetes | 0 | 0 | 0 | 0 | 0 | 0 |
| Hypertension | 0 | 0 | 0 | 0 | 0 | 0 |
| Chronic kidney disease | 0 | 0 | 0 | 0 | 0 | 0 |
| Acute kidney injury | 0 | 0 | 0 | 0 | 0 | 0 |
| Coronary artery disease | 0 | 0 | 0 | 0 | 0 | 0 |
| Cardiovascular disease | 0 | 0 | 0 | 0 | 0 | 0 |
| Malignancy | 0 | 0 | 0 | 0 | 0 | 0 |
| Medication history of anti-diabetic medication | 0 | 0 | 0 | 0 | 0 | 0 |
| Anti-hypertensive medication | 0 | 0 | 0 | 0 | 0 | 0 |
| Drug usage history within 14 days of NSAID | 0 | 0 | 0 | 0 | 0 | 0 |
| Drug usage history within 90 days of NSAID | 0 | 0 | 0 | 0 | 0 | 0 |
| Drug usage history within 14 days of diuretics | 0 | 0 | 0 | 0 | 0 | 0 |
| Drug usage history within 90 days of diuretics | 0 | 0 | 0 | 0 | 0 | 0 |
| Drug usage history within 14 days of renin-angiotensin-aldosterone system blockades | 0 | 0 | 0 | 0 | 0 | 0 |
| Drug usage history within 90 days of renin-angiotensin-aldosterone system blockades | 0 | 0 | 0 | 0 | 0 | 0 |
| Drug usage history within 14 days of aspirin | 0 | 0 | 0 | 0 | 0 | 0 |
| Drug usage history within 90 days of aspirin | 0 | 0 | 0 | 0 | 0 | 0 |
| Drug usage history within 14 days of clopidogrel | 0 | 0 | 0 | 0 | 0 | 0 |
| Drug usage history within 90 days of clopidogrel | 0 | 0 | 0 | 0 | 0 | 0 |
| Drug usage history within 14 days of ezetimibe | 0 | 0 | 0 | 0 | 0 | 0 |
| Drug usage history within 90 days of ezetimibe | 0 | 0 | 0 | 0 | 0 | 0 |
| Drug usage history within 14 days of fenofibrate | 0 | 0 | 0 | 0 | 0 | 0 |
| Drug usage history within 90 days of fenofibrate | 0 | 0 | 0 | 0 | 0 | 0 |
| Drug usage history within 14 days of immunosuppressants | 0 | 0 | 0 | 0 | 0 | 0 |
| Drug usage history within 90 days of immunosuppressants | 0 | 0 | 0 | 0 | 0 | 0 |
| Drug usage history within 14 days of direct oral anticoagulant | 0 | 0 | 0 | 0 | 0 | 0 |
| Drug usage history within 90 days of direct oral anticoagulant | 0 | 0 | 0 | 0 | 0 | 0 |
| Drug usage history within 14 days of low-molecular weight heparin | 0 | 0 | 0 | 0 | 0 | 0 |
| Drug usage history within 90 days of low-molecular weight heparin | 0 | 0 | 0 | 0 | 0 | 0 |
| Drug usage history within 14 days statin | 0 | 0 | 0 | 0 | 0 | 0 |
| Drug usage history within 90 days statin | 0 | 0 | 0 | 0 | 0 | 0 |
| Drug usage history within 14 days steroid | 0 | 0 | 0 | 0 | 0 | 0 |
| Drug usage history within 90 days steroid | 0 | 0 | 0 | 0 | 0 | 0 |
| Drug usage history within 14 days warfarin | 0 | 0 | 0 | 0 | 0 | 0 |
| Drug usage history within 90 days warfarin | 0 | 0 | 0 | 0 | 0 | 0 |
| eGFR category | 0 | 0 | 0 | 0 | 0 | 0 |
| Dipstick urine test results for presence of urine albumin | 0 | 0 | 0 | 0 | 0 | 0 |
| Dipstick urine test results for presence of urine RBC | 10248 | 19.959 | 1375 | 2.92 | 72 | 0.587 |
| Age | 0 | 0 | 0 | 0 | 0 | 0 |
| Systolic BP | 2017 | 3.928 | 55 | 0.117 | 137 | 1.118 |
| Diastolic BP | 2018 | 3.93 | 55 | 0.117 | 137 | 1.118 |
| Heart rate | 32961 | 64.195 | 55 | 0.117 | 137 | 1.118 |
| Height | 32038 | 62.398 | 159 | 0.338 | 216 | 1.762 |
| Weight | 32022 | 62.366 | 129 | 0.274 | 105 | 0.857 |
| Body mass index | 1049 | 2.043 | 210 | 0.446 | 232 | 1.892 |
| Duration of admission before operation | 0 | 0 | 0 | 0 | 0 | 0 |
| Estimated operation duration | 0 | 0 | 0 | 0 | 0 | 0 |
| Hemoglobin | 0 | 0 | 0 | 0 | 0 | 0 |
| Hematocrit | 12 | 0.023 | 0 | 0 | 0 | 0 |
| Platelet count | 232 | 0.452 | 0 | 0 | 0 | 0 |
| White blood cell | 6 | 0.012 | 0 | 0 | 0 | 0 |
| Erythrocyte sedimentation rate | 25870 | 50.385 | 19752 | 41.943 | 3645 | 29.733 |
| Neutrophil count | 10025 | 19.525 | 46881 | 99.55 | 102 | 0.832 |
| CRP | 15757 | 30.688 | 11666 | 24.772 | 1484 | 12.105 |
| Sodium | 0 | 0 | 0 | 0 | 0 | 0 |
| Potassium | 1 | 0.002 | 0 | 0 | 0 | 0 |
| Chloride | 74 | 0.144 | 1 | 0.002 | 0 | 0 |
| Total CO2 | 13739 | 26.758 | 13317 | 28.278 | 6112 | 49.857 |
| Blood urea nitrogen | 0 | 0 | 1 | 0.002 | 0 | 0 |
| Creatinine | 0 | 0 | 0 | 0 | 0 | 0 |
| eGFR (CKD-EPI) | 0 | 0 | 0 | 0 | 0 | 0 |
| Total protein | 15 | 0.029 | 1 | 0.002 | 2 | 0.016 |
| Albumin | 0 | 0 | 0 | 0 | 0 | 0 |
| Cholesterol | 16 | 0.031 | 1005 | 2.134 | 364 | 2.969 |
| Low-density lipoprotein | 46319 | 90.211 | 42059 | 89.311 | 9940 | 81.090 |
| High-density lipoprotein | 44622 | 86.906 | 42032 | 89.253 | 9796 | 79.915 |
| Triglyceride | 44661 | 86.982 | 41642 | 88.425 | 9775 | 79.744 |
| Calcium | 11 | 0.021 | 4 | 0.008 | 30 | 0.245 |
| Phosphate | 12 | 0.023 | 15 | 0.032 | 29 | 0.237 |
| Uric acid | 257 | 0.501 | 1019 | 2.164 | 267 | 2.178 |
| Alanine aminotransferase | 2645 | 5.151 | 2188 | 4.646 | 0 | 0 |
| Aspartate aminotransferase | 2 | 0.004 | 2188 | 4.646 | 0 | 0 |
| Alkaline phosphatase | 13 | 0.025 | 1 | 0.002 | 0 | 0 |
| Hemoglobin A1c | 37844 | 73.705 | 26469 | 56.206 | 6769 | 55.221 |
| Parathyroid hormone | 51031 | 99.388 | 46666 | 99.093 | 11582 | 94.485 |
| Urine protein-to-creatinine ratio | 50413 | 98.185 | 46221 | 98.148 | 12258 | 100 |
| Total bilirubin | 6 | 0.012 | 1 | 0.002 | 0 | 0 |
| Glucose | 433 | 0.843 | 786 | 1.669 | 254 | 2.072 |
| Prothrombin time (INR) | 160 | 0.312 | 47093 | 100 | 80 | 0.653 |
|  |  |  |  |  |  |  |

Abbreviations: EVC= External validation cohort; ASA= American Society of Anesthesiologists; NYHA= New York Heart Association; COPD= Chronic obstructive pulmonary disease; NSAID= Non-steroidal anti-inflammatory drug; eGFR= Estimated glomerular filtration rate; RBC= Red blood cell; BP= Blood pressure; CRP= C-reactive protein; CKD-EPI= Chronic kidney disease epidemiology collaboration; INR= International normalized ratio
