# Supplementary material for: A deep-learning algorithm using real-time collected intraoperative vital sign signals for predicting acute kidney injury after major non-cardiac surgeries: A modelling study
Source: PLoS Med. 2025 Apr 29;22(4):e1004566. doi: 10.1371/journal.pmed.1004566 (PMC12040160; doi:10.1371/journal.pmed.1004566)
Supplement: S2 Table — (DOCX) [file pmed.1004566.s003.docx]

**S2 Table. Characteristics of the intraoperative vital sign signal derived variables.**

| **Intraoperative vital sign-derived variables** | **Discovery cohort (N = 51,345)** | **EVC 1 (N = 47,093)** | **EVC 2 (N = 12,259)** |
| --- | --- | --- | --- |
| Systolic BP measurement duration (min) | 159.0 [103.0, 232.0] | 113.0 [70.0, 171.0] | 88.0 [65.0, 136.0] |
| Diastolic BP measurement duration (min) | 159.0 [103.0, 232.0] | 113.0 [70.0, 171.0] | 88.0 [65.0, 136.0] |
| Heart rate measurement duration (min) | 156.0 [103.0, 230.0] | 112.0 [73.0, 161.0] | 88.0 [64.0, 136.0] |
| Total duration of mean BP <65 mmHg (min) | 5.0 [0.0, 18.0] | 0.0 [0.0, 15.0] | 0.0 [0.0, 11.0] |
| Duration of mean BP <65 mmHg (min) | 4.0 [0.0, 9.0] | 0.0 [0.0, 8.0] | 0.0 [0.0, 4.0] |
| Duration of heart rate <60/min (min) | 6.0 [0.0, 57.0] | 7.0 [0.0, 43.0] | 12.0 [0.0, 52.0] |
| Duration of heart rate > 100/min (min) | 0.0 [0.0, 3.0] | 0.0 [0.0, 0.0] | 0.0 [0.0, 0.0] |
| SD of systolic BP | 14.2 [10.5, 18.2] | 9.9 [6.5, 14.0] | 13.6 [9.3, 18.4] |
| SD of diastolic BP | 8.1 [6.2, 10.2] | 6.8 [4.5, 9.3] | 7.7 [5.4, 10.6] |
| SD of heart rate | 6.4 [4.6, 8.6] | 5.8 [4.0, 7.9] | 5.6 [3.7, 8.2] |
| CV of systolic BP | 0.1 [0.1, 0.2] | 0.1 [0.1, 0.1] | 0.1 [0.1, 0.2] |
| CV of diastolic BP | 0.1 [0.1, 0.2] | 0.1 [0.1, 0.1] | 0.1 [0.1, 0.2] |
| CV of heart rate | 0.1 [0.1, 0.1] | 0.1 [0.1, 0.1] | 0.1 [0.1, 0.1] |
| ARV of systolic BP | 3.0 [2.2, 4.1] | 1.2 [0.4, 2.2] | 2.5 [1.6, 3.5] |
| ARV of diastolic BP | 1.9 [1.3, 2.6] | 0.9 [0.3, 1.6] | 1.5 [1.0, 2.1] |
| ARV of heart rate | 1.2 [0.8, 1.8] | 0.9 [0.6, 1.2] | 0.8 [0.5, 1.2] |
| VIM of systolic BP | 0.0 [0.0, 0.0] | 0.0 [0.0, 0.0] | 0.3 [0.3, 0.4] |
| VIM of diastolic BP | 0.0 [0.0, 0.0] | 0.0 [0.0, 0.0] | 0.1 [0.1, 0.1] |
| VIM of heart rate | 0.0 [0.0, 0.0] | 0.0 [0.0, 0.0] | 0.0 [0.0, 0.0] |
| Maximum drop in systolic BP | 28.0 [19.0, 39.0] | 20.0 [11.0, 31.0] | 29.0 [18.0, 44.0] |
| Maximum drop in diastolic BP | 18.0 [12.0, 25.0] | 14.0 [8.0, 21.0] | 17.0 [11.0, 27.0] |
| Maximum drop in heart rate | 12.0 [8.0, 18.0] | 12.0 [7.0, 19.0] | 9.0 [5.0, 17.0] |
| Maximum rise in systolic BP | 32.0 [22.0, 46.0] | 20.0 [11.0, 33.0] | 30.0 [17.0, 47.0] |
| Maximum rise in diastolic BP | 20.0 [14.0, 28.0] | 14.0 [8.0, 22.0] | 17.0 [11.0, 28.0] |
| Maximum rise in heart rate | 18.0 [10.0, 26.0] | 12.0 [8.0, 19.0] | 12.0 [7.0, 19.5] |

Data are presented as median [interquartile range] for continuous variables and n (%) for categorical variables.

Abbreviations: EVC= External validation cohort; BP= Blood pressure; SD= Standard deviation; CV= Coefficient of variation; ARV= Average real variability; VIM= Variability independent of the mean.
