# Supplementary material for: A deep-learning algorithm using real-time collected intraoperative vital sign signals for predicting acute kidney injury after major non-cardiac surgeries: A modelling study
Source: PLoS Med. 2025 Apr 29;22(4):e1004566. doi: 10.1371/journal.pmed.1004566 (PMC12040160; doi:10.1371/journal.pmed.1004566)
Supplement: S3 Table — (DOCX) [file pmed.1004566.s004.docx]

**S3 Table. Performance of different model architectures for postoperative AKI risk prediction.**

| Outcome | Hospital | Model | AUROC | p-value (vs. DL-IVSS_PCFs 11) |
| --- | --- | --- | --- | --- |
| PO AKI | Developmental cohort | DL-IVSS_PCFs 11 (EfficientNet-b3) | 0.765  (0.745, 0.785) | - |
|  |  | LSTM_PCFs 11 | 0.728  (0.706, 0.749) | <0.001 |
|  |  | ResNet34_PCFs 11 | 0.763  (0.743, 0.784) | 0.547 |
|  |  | ResNet50_PCFs 11 | 0.765  (0.744, 0.785) | 0.929 |
|  |  | EfficientNet-b5_PCFs 11 | 0.731  (0.710, 0.753) | <0.001 |

Performance metrics are presented as the calculated values with 95% confidence intervals in parentheses.

Abbreviations: PO AKI= Postoperative acute kidney injury; AUROC= Area under the receiver operating characteristic curve; DL-IVSS_PCFs 11= A deep-learning algorithm (EfficientNet-b3) leveraging time-series intraoperative vital sign signals and preoperative clinical features 11; LSTM_PCFs 11= A LSTM leveraging time-series intraoperative vital sign signals and preoperative clinical features 11; LSTM_PCFs 11= A LSTM leveraging time-series intraoperative vital sign signals and preoperative clinical features 11; ResNet34_PCFs 11= A ResNet34 leveraging time-series intraoperative vital sign signals and preoperative clinical features 11; ResNet50_PCFs 11= A ResNet50 leveraging time-series intraoperative vital sign signals and preoperative clinical features 11; EfficientNet-b5_PCFs 11= A EfficientNet-b5 leveraging time-series intraoperative vital sign signals and preoperative clinical features 11
