# Supplementary material for: A deep-learning algorithm using real-time collected intraoperative vital sign signals for predicting acute kidney injury after major non-cardiac surgeries: A modelling study
Source: PLoS Med. 2025 Apr 29;22(4):e1004566. doi: 10.1371/journal.pmed.1004566 (PMC12040160; doi:10.1371/journal.pmed.1004566)
Supplement: S4 Table — (DOCX) [file pmed.1004566.s005.docx]

**S4 Table. Characteristics of the study variables according to the presence of postoperative AKI in each study hospital.**

|  | **Developmental cohort** | | **EVC 1** | | **EVC 2** | |
| --- | --- | --- | --- | --- | --- | --- |
|  | **No AKI (N = 48,157)** | **PO-AKI (N = 3,188)** | **No AKI (N = 44,574)** | **PO-AKI (N = 2,519)** | **No AKI (N = 11,679)** | **PO-AKI (N = 579)** |
| **Preoperative risk factors** |  |  |  |  |  |  |
| Age (years) | 59.0 [48.0, 69.0] | 64.0 [54.0, 72.0] | 61.0 [50.0, 70.0] | 67.0 [57.0, 74.0] | 65.0 [54.0, 73.0] | 70.0 [59.0, 76.0] |
| <40 | 6706 (13.9) | 213 (6.7) | 5017 (11.3) | 164 (6.5) | 1090 (9.3) | 36 (6.2) |
| ≥40 and <60 | 17501 (36.3) | 971 (30.5) | 15509 (34.8) | 591 (23.5) | 2966 (25.4) | 114 (19.7) |
| ≥60 and <80 | 22344 (46.4) | 1788 (56.1) | 22051 (49.5) | 1563 (62.0) | 6819 (58.4) | 343 (59.2) |
| ≥80 | 1606 (3.3) | 216 (6.8) | 1997 (4.5) | 201 (8.0) | 804 (6.9) | 86 (14.9) |
| eGFR (mL/min per 1.73 m^2^) | 90.2 [76.8, 102.1] | 83.0 [64.3, 98.0] | 93.5 [82.7, 103.3] | 87.4 [70.0, 99.0] | 91.7 [81.0, 101.4] | 83.3 [59.6, 99.0] |
| ≥60 | 45190 (93.8) | 2553 (80.1) | 42358 (95.0) | 2105 (83.6) | 10873 (93.1) | 432 (74.6) |
| ≥45 and <60 | 2380 (4.9) | 395 (12.4) | 1655 (3.7) | 229 (9.1) | 574 (4.9) | 74 (12.8) |
| ≥30 and <45 | 475 (1.0) | 164 (5.1) | 444 (1.0) | 121 (4.8) | 192 (1.6) | 44 (7.6) |
| ≥15 and <30 | 112 (0.2) | 76 (2.4) | 117 (0.3) | 64 (2.5) | 40 (0.3) | 29 (5.0) |
| Dipstick albuminuria (urine albumin ≥1+) | 4961 (10.3) | 777 (24.4) | 2775 (6.2) | 454 (18.0) | 1188 (10.2) | 155 (26.8) |
| Sex |  |  |  |  |  |  |
| Female | 22061 (45.8) | 1076 (33.8) | 22355 (50.2) | 812 (32.2) | 6767 (57.9) | 261 (45.1) |
| Male | 26096 (54.2) | 2112 (66.2) | 22219 (49.8) | 1707 (67.8) | 4912 (42.1) | 318 (54.9) |
| Expected surgical duration (hours) | 15.0 [10.0, 20.0] | 15.0 [10.0, 25.0] | 15.0 [10.0, 20.0] | 15.0 [10.0, 20.0] | 10.0 [10.0, 15.0] | 10.0 [10.0, 15.0] |
| Emergency operation | 3446 (7.2) | 326 (10.2) | 602 (1.4) | 100 (4.0) | 207 (1.8) | 23 (4.0) |
| Diabetes mellitus | 5646 (11.7) | 686 (21.5) | 7132 (16.0) | 634 (25.2) | 2238 (19.2) | 193 (33.3) |
| RAAS blockade use | 2967 (6.2) | 405 (12.7) | 1804 (4.0) | 165 (6.6) | 1652 (14.1) | 139 (24.0) |
| Hypoalbuminemia (<3.5 g/dL) | 4514 (9.4) | 861 (27.0) | 10688 (24.0) | 1001 (39.7) | 2127 (18.2) | 258 (44.6) |
| Anemia (<12 g/dL for female, 13 g/dL for male) | 13539 (28.1) | 1577 (49.5) | 17288 (38.8) | 1372 (54.5) | 5280 (45.2) | 415 (71.7) |
| Hyponatremia (<135 mEq/L) | 1230 (2.6) | 261 (8.2) | 929 (2.1) | 116 (4.6) | 642 (5.5) | 66 (11.4) |
| **Intraoperative vital sign-derived variables** |  |  |  |  |  |  |
| Systolic BP measurement duration (min) | 156.0 [102.0, 227.0] | 222.0 [129.0, 339.2] | 111.0 [69.0, 168.0] | 142.0 [87.0, 233.0] | 88.0 [64.0, 135.5] | 108.0 [72.0, 180.0] |
| Diastolic BP measurement duration (min) | 156.0 [102.0, 227.0] | 222.0 [129.0, 339.2] | 111.0 [69.0, 168.0] | 142.0 [87.0, 233.0] | 88.0 [64.0, 135.5] | 108.0 [72.0, 180.0] |
| Heart rate measurement duration (min) | 156.0 [101.0, 224.0] | 221.0 [130.0, 336.0] | 110.5 [72.0, 159.0] | 137.0 [93.5, 217.0] | 88.0 [64.0, 136.0] | 108.0 [72.0, 180.0] |
| Total duration of mean BP <65 mmHg (min) | 5.0 [0.0, 17.0] | 15.0 [2.0, 50.0] | 0.0 [0.0, 14.0] | 1.0 [0.0, 29.0] | 0.0 [0.0, 8.0] | 4.0 [0.0, 20.0] |
| Duration of mean BP <65 mmHg (min) | 3.0 [0.0, 8.0] | 6.0 [0.0, 16.0] | 0.0 [0.0, 8.0] | 0.0 [0.0, 19.0] | 0.0 [0.0, 4.0] | 4.0 [0.0, 8.0] |
| Duration of heart rate <60/min (min) | 6.0 [0.0, 58.0] | 3.0 [0.0, 45.2] | 8.0 [0.0, 43.0] | 4.0 [0.0, 39.0] | 12.0 [0.0, 52.0] | 4.0 [0.0, 48.0] |
| Duration of heart rate > 100/min (min) | 0.0 [0.0, 3.0] | 0.0 [0.0, 12.2] | 0.0 [0.0, 0.0] | 0.0 [0.0, 4.0] | 0.0 [0.0, 0.0] | 0.0 [0.0, 4.0] |
| SD of systolic BP | 14.0 [10.4, 18.1] | 15.7 [12.4, 19.4] | 9.8 [6.5, 13.9] | 11.2 [7.6, 15.3] | 13.5 [9.2, 18.4] | 15.2 [11.1, 19.6] |
| SD of diastolic BP | 8.1 [6.2, 10.2] | 8.5 [6.8, 10.5] | 6.8 [4.5, 9.2] | 7.2 [5.0, 9.6] | 7.7 [5.4, 10.6] | 8.2 [5.9, 11.7] |
| SD of heart rate | 6.4 [4.5, 8.6] | 7.0 [5.1, 9.2] | 5.7 [4.0, 7.9] | 6.5 [4.6, 8.9] | 5.6 [3.7, 8.2] | 6.1 [3.7, 8.9] |
| CV of systolic BP | 0.1 [0.1, 0.2] | 0.1 [0.1, 0.2] | 0.1 [0.1, 0.1] | 0.1 [0.1, 0.1] | 0.1 [0.1, 0.2] | 0.1 [0.1, 0.2] |
| CV of diastolic BP | 0.1 [0.1, 0.2] | 0.1 [0.1, 0.2] | 0.1 [0.1, 0.1] | 0.1 [0.1, 0.1] | 0.1 [0.1, 0.2] | 0.1 [0.1, 0.2] |
| CV of heart rate | 0.1 [0.1, 0.1] | 0.1 [0.1, 0.1] | 0.1 [0.1, 0.1] | 0.1 [0.1, 0.1] | 0.1 [0.1, 0.1] | 0.1 [0.1, 0.1] |
| ARV of systolic BP | 3.0 [2.2, 4.1] | 3.1 [2.2, 4.3] | 1.3 [0.4, 2.2] | 0.7 [0.3, 1.9] | 2.4 [1.6, 3.5] | 2.8 [1.8, 3.8] |
| ARV of diastolic BP | 1.9 [1.4, 2.6] | 1.8 [1.3, 2.5] | 0.9 [0.3, 1.6] | 0.4 [0.2, 1.2] | 1.4 [1.0, 2.1] | 1.5 [1.1, 2.3] |
| ARV of heart rate | 1.2 [0.8, 1.8] | 1.0 [0.7, 1.6] | 0.9 [0.6, 1.2] | 0.9 [0.6, 1.3] | 0.8 [0.5, 1.2] | 0.8 [0.5, 1.2] |
| VIM of systolic BP | 0.0 [0.0, 0.0] | 0.0 [0.0, 0.0] | 0.0 [0.0, 0.0] | 0.0 [0.0, 0.0] | 0.3 [0.3, 0.4] | 0.4 [0.3, 0.4] |
| VIM of diastolic BP | 0.0 [0.0, 0.0] | 0.0 [0.0, 0.0] | 0.0 [0.0, 0.0] | 0.0 [0.0, 0.0] | 0.1 [0.1, 0.1] | 0.1 [0.1, 0.1] |
| VIM of heart rate | 0.0 [0.0, 0.0] | 0.0 [0.0, 0.0] | 0.0 [0.0, 0.0] | 0.0 [0.0, 0.0] | 0.0 [0.0, 0.0] | 0.0 [0.0, 0.0] |
| Maximum drop in systolic BP | 27.0 [19.0, 39.0] | 34.0 [24.0, 48.0] | 20.0 [11.0, 31.0] | 23.0 [14.0, 35.0] | 29.0 [18.0, 44.0] | 34.0 [20.0, 52.0] |
| Maximum drop in diastolic BP | 18.0 [12.0, 25.0] | 20.0 [14.0, 28.0] | 14.0 [8.0, 21.0] | 15.0 [9.0, 23.0] | 17.0 [11.0, 27.0] | 20.0 [12.0, 32.0] |
| Maximum drop in heart rate | 12.0 [8.0, 18.0] | 14.0 [8.0, 20.0] | 12.0 [7.0, 19.0] | 14.0 [8.0, 22.0] | 9.0 [5.0, 17.0] | 11.0 [6.0, 18.0] |
| Maximum rise in systolic BP | 31.0 [22.0, 46.0] | 38.0 [26.0, 52.0] | 20.0 [11.0, 32.0] | 22.0 [12.0, 35.0] | 30.0 [17.0, 46.0] | 34.0 [21.0, 53.0] |
| Maximum rise in diastolic BP | 20.0 [14.0, 28.0] | 22.0 [16.0, 30.0] | 14.0 [8.0, 22.0] | 14.0 [8.0, 23.0] | 17.0 [10.0, 28.0] | 21.0 [13.0, 32.0] |
| Maximum rise in heart rate | 18.0 [10.0, 26.0] | 18.0 [12.0, 26.0] | 12.0 [8.0, 19.0] | 14.0 [9.0, 21.0] | 12.0 [7.0, 19.0] | 12.0 [7.0, 21.0] |

Data are presented as median [interquartile range] for continuous variables and n (%) for categorical variables.

Abbreviations: AKI= Acute kidney injury; PO-AKI= Postoperative acute kidney injury; EVC= External validation cohort; RAAS= Renin-angiotensin-aldosterone system; BP= Blood pressure; SD= Standard deviation; CV= Coefficient of variation; ARV= Average real variability; VIM= Variability independent of the mean
