# Supplementary material for: A deep-learning algorithm using real-time collected intraoperative vital sign signals for predicting acute kidney injury after major non-cardiac surgeries: A modelling study
Source: PLoS Med. 2025 Apr 29;22(4):e1004566. doi: 10.1371/journal.pmed.1004566 (PMC12040160; doi:10.1371/journal.pmed.1004566)
Supplement: S5 Table — (DOCX) [file pmed.1004566.s006.docx]

**S5 Table. Discriminative performances for postoperative AKI risk by intraoperative vital sign signals and additional summary-level vital sign information.**

| **Outcome** | **Hospital** | **Method** | **AUROC** | **p-value (vs. SPARK)** | **p-value (vs. preceding model)** | **Balanced Accuracy** | **NPV (Spec 0.95)** | **PPV (Sens 0.95)** |
| --- | --- | --- | --- | --- | --- | --- | --- | --- |
| PO AKI | Developmental cohort | DL-IVSS_PCVSFs 28 | 0.760  (0.744, 0.772) | <0.001 | 0.516 | 0.700 (0.681, 0.719) | 0.957 (0.951, 0.962) | 0.076 (0.068, 0.084) |
|  | EVC 1 | DL-IVSS_PCVSFs 28 | 0.713  (0.702, 0.723) | <0.001 | 0.486 | 0.658 (0.648, 0.668) | 0.953 (0.951, 0.955) | 0.071 (0.069, 0.074) |
|  | EVC 2 | DL-IVSS_PCVSFs 28 | 0.598  (0.574, 0.612) | <0.001 | <0.001 | 0.574 (0.553, 0.597) | 0.956 (0.952, 0.960) | 0.048 (0.044, 0.053) |
| Critical AKI | Developmental cohort | DL-IVSS_PCVSFs 28 | 0.753  (0.702, 0.787) | 0.104 | 0.005 | 0.711 (0.664, 0.758) | 0.993 (0.991, 0.995) | 0.012 (0.009, 0.016) |
|  | EVC 1 | DL-IVSS_PCVSFs 28 | 0.714  (0.687, 0.736) | 0.26 | 0.038 | 0.663 (0.640, 0.689) | 0.991 (0.990, 0.992) | 0.016 (0.014, 0.017) |
|  | EVC 2 | DL-IVSS_PCVSFs 28 | 0.502  (0.462, 0.537) | <0.001 | <0.001 | 0.482 (0.439, 0.523) | 0.991 (0.989, 0.993) | 0.009 (0.007, 0.011) |

Performance metrics are presented as the calculated values with 95% confidence intervals in parentheses. The "p-value (vs. preceding model)” column represents the p-value comparing the performance of the DL-IVSS_PCVSFs 28 model with the DL-IVSS_PCFs 11 model. The "NPV (Spec 0.95)" column represents the negative predictive value (NPV) when a specificity threshold of 95% was applied. The "PPV (Sens 0.95)" column represents the positive predictive value (PPV) when a sensitivity threshold of 95% was applied.

Abbreviations: PO-AKI= Postoperative acute kidney injury; EVC= External validation cohort; AUROC= Area under the receiver operating characteristic curve; PPV= Positive predictive value; NPV= Negative predictive value; DL-IVSS_PCSFs 28= A deep-learning algorithm leveraging time-series intraoperative vital sign signals and preoperative clinical and vital sign features 28
