# Supplementary material for: A deep-learning algorithm using real-time collected intraoperative vital sign signals for predicting acute kidney injury after major non-cardiac surgeries: A modelling study
Source: PLoS Med. 2025 Apr 29;22(4):e1004566. doi: 10.1371/journal.pmed.1004566 (PMC12040160; doi:10.1371/journal.pmed.1004566)
Supplement: S6 Table — (DOCX) [file pmed.1004566.s007.docx]

**S6 Table. Discriminative performances for postoperative AKI risk by preoperative ML model.**

| **Outcome** | **Hospital** | **Method** | **AUROC** | **p-value (vs.  SPARK)** | **Balanced Accuracy** | **NPV  (Spec  0.95)** | **PPV  (Sens  0.95)** |
| --- | --- | --- | --- | --- | --- | --- | --- |
| PO-AKI | Developmental  cohort | preOp_ML | 0.781  (0.765-0.794) | <0.001 | 0.726 (0.708-0.742) | 0.955 (0.949-0.961) | 0.076 (0.067-0.085) |
|  |  |  |  |  |  |  |  |
|  | EVC 1 | preOp_ML | 0.760 (0.753-0.769) | <0.001 | 0.697 (0.687-0.706) | 0.963 (0.962-0.965) | 0.062 (0.059-0.064) |
|  |  |  |  |  |  |  |  |
|  | EVC 2 | preOp_ML | 0.769  (0.757-0.79) | 0.008 | 0.700 (0.680-0.720) | 0.966 (0.962-0.969) | 0.055 (0.051-0.060) |
|  |  |  |  |  |  |  |  |
| Critical AKI | Developmental  cohort | preOp_ML | 0.838  (0.811-0.860) | 0.012 | 0.763 (0.721-0.804) | 0.993 (0.991-0.996) | 0.015 (0.011-0.019) |
|  |  |  |  |  |  |  |  |
|  | EVC 1 | preOp_ML | 0.822  (0.807-0.833) | <0.001 | 0.759 (0.740-0.776) | 0.995 (0.994-0.996) | 0.012 (0.011-0.013) |
|  |  |  |  |  |  |  |  |
|  | EVC 2 | preOp_ML | 0.796  (0.761-0.821) | 0.092 | 0.735 (0.690-0.779) | 0.994 (0.993-0.996) | 0.012 (0.010-0.014) |
|  |  |  |  |  |  |  |  |

Performance metrics are presented as the calculated values with 95% confidence intervals in parentheses. The "NPV (Spec 0.95)" column represents the negative predictive value (NPV) when a specificity threshold of 95% was applied. The "PPV (Sens 0.95)" column represents the positive predictive value (PPV) when a sensitivity threshold of 95% was applied.

Abbreviations: PO-AKI= Postoperative acute kidney injury; EVC= External validation cohort; AUROC= Area under the receiver operating characteristic curve; PPV= Positive predictive value; NPV= Negative predictive value; preOp_ML= Preoperative machine learning model
