# Supplementary material for: A deep-learning algorithm using real-time collected intraoperative vital sign signals for predicting acute kidney injury after major non-cardiac surgeries: A modelling study
Source: PLoS Med. 2025 Apr 29;22(4):e1004566. doi: 10.1371/journal.pmed.1004566 (PMC12040160; doi:10.1371/journal.pmed.1004566)
Supplement: S7 Table — (DOCX) [file pmed.1004566.s008.docx]

**S7 Table. Discriminative performances for postoperative AKI risk by ensemble models and additional summary-level vital sign information.**

| **Outcome** | **Hospital** | **Method** | **AUROC** | **p-value (vs. SPARK)** | **p-value (vs. preceding model)** | **Balanced Accuracy** | **NPV (Spec 0.95)** | **PPV (Sens 0.95)** |
| --- | --- | --- | --- | --- | --- | --- | --- | --- |
| PO AKI | Developmental cohort | Ensemble_PCVSFs 28 | 0.796  (0.774, 0.805) | <0.001 | 0.975 | 0.725 (0.704, 0.744) | 0.959 (0.954, 0.965) | 0.080 (0.072, 0.088) |
|  | EVC 1 | Ensemble_PCVSFs 28 | 0.766  (0.761, 0.772) | <0.001 | 0.015 | 0.700 (0.691, 0.71) | 0.961 (0.959, 0.962) | 0.068 (0.065, 0.071) |
|  | EVC 2 | Ensemble_PCVSFs 28 | 0.750  (0.728, 0.764) | 0.546 | <0.001 | 0.689 (0.668, 0.708) | 0.964 (0.960, 0.967) | 0.050 (0.046, 0.054) |
| Critical AKI | Developmental cohort | Ensemble_PCVSFs 28 | 0.846  (0.801, 0.872) | 0.008 | 0.736 | 0.784 (0.741, 0.824) | 0.994 (0.992, 0.997) | 0.015 (0.011, 0.019) |
|  | EVC 1 | Ensemble_PCVSFs 28 | 0.825  (0.813, 0.836) | <0.001 | <0.001 | 0.757 (0.737, 0.776) | 0.994 (0.993, 0.995) | 0.015 (0.014, 0.017) |
|  | EVC 2 | Ensemble_PCVSFs 28 | 0.740  (0.702, 0.764) | 0.606 | 0.001 | 0.692 (0.648, 0.738) | 0.994 (0.992, 0.995) | 0.010  (0.008, 0.012) |

Performance metrics are presented as the calculated values with 95% confidence intervals in parentheses. The "p-value (vs. preceding model)” column represents the p-value comparing the performance of the Ensemble_PCVSFs 28 model with the Ensemble_PCFs 11 model. The "NPV (Spec 0.95)" column represents the negative predictive value (NPV) when a specificity threshold of 95% was applied. The "PPV (Sens 0.95)" column represents the positive predictive value (PPV) when a sensitivity threshold of 95% was applied.

Abbreviations: PO-AKI= Postoperative acute kidney injury; EVC= External validation cohort; AUROC= Area under the receiver operating characteristic curve; PPV= Positive predictive value; NPV= Negative predictive value; Ensemble_PCVSFs 28= A ensemble model combining preOp_ML and DL-IVSS_PCVSFs 28
