# Supplementary material for: A deep-learning algorithm using real-time collected intraoperative vital sign signals for predicting acute kidney injury after major non-cardiac surgeries: A modelling study
Source: PLoS Med. 2025 Apr 29;22(4):e1004566. doi: 10.1371/journal.pmed.1004566 (PMC12040160; doi:10.1371/journal.pmed.1004566)
Supplement: S8 Table — (DOCX) [file pmed.1004566.s009.docx]

**S8 Table. Model performance in each study data when sensitivity 95% threshold was applied.**

| **Output** | **Model** | **Hospital** | **Total positive** | **Predicted as positive** | **TP** | **FP** | **TN** | **FN** |
| --- | --- | --- | --- | --- | --- | --- | --- | --- |
| PO-AKI | DL-IVSS_PCFs 11 | Developmental cohort for threshold | 319 | 4271 | 303 | 3968 | 847 | 16 |
|  |  | Developmental cohort for test | 319 | 4242 | 308 | 3934 | 882 | 11 |
|  |  | EVC 1 | 2519 | 38820 | 2374 | 36446 | 8128 | 145 |
|  |  | EVC 2 | 579 | 10315 | 560 | 9755 | 1924 | 19 |
|  | Ensemble_PCFs 11 | Developmental cohort for threshold | 319 | 4080 | 303 | 3777 | 1038 | 16 |
|  |  | Developmental cohort for test | 319 | 4057 | 307 | 3750 | 1066 | 12 |
|  |  | EVC 1 | 2519 | 38967 | 2419 | 36548 | 8026 | 100 |
|  |  | EVC 2 | 579 | 10086 | 565 | 9521 | 2158 | 14 |
| Critical AKI | DL-IVSS_PCFs 11 | Developmental cohort for threshold | 53 | 3830 | 50 | 3780 | 1301 | 3 |
|  |  | Developmental cohort for test | 53 | 3827 | 51 | 3776 | 1306 | 2 |
|  |  | EVC 1 | 448 | 35137 | 422 | 34715 | 11930 | 26 |
|  |  | EVC 2 | 111 | 8493 | 104 | 8389 | 3758 | 7 |
|  | Ensemble_PCFs 11 | Developmental cohort for threshold | 53 | 3022 | 50 | 2972 | 2109 | 3 |
|  |  | Developmental cohort for test | 53 | 2994 | 47 | 2947 | 2135 | 6 |
|  |  | EVC 1 | 448 | 30945 | 422 | 30523 | 16122 | 26 |
|  |  | EVC 2 | 111 | 7110 | 103 | 7007 | 5140 | 8 |

Abbreviations: PO-AKI= Postoperative acute kidney injury; EVC= External validation cohort; TP= True positive; FP= False positive; TN= True negative; FN= False negative; DL-IVSS_PCFs 11= A deep-learning algorithm leveraging time-series intraoperative vital sign signals and preoperative clinical features 11; Ensemble_PCFs 11= A ensemble model combining preOp_ML and DL-IVSS_PCFs 11
