# Supplementary material for: A deep-learning algorithm using real-time collected intraoperative vital sign signals for predicting acute kidney injury after major non-cardiac surgeries: A modelling study
Source: PLoS Med. 2025 Apr 29;22(4):e1004566. doi: 10.1371/journal.pmed.1004566 (PMC12040160; doi:10.1371/journal.pmed.1004566)
Supplement: S9 Table — (DOCX) [file pmed.1004566.s010.docx]

**S9 Table. Model performance in each study data when specificity 95% threshold was applied.**

| **Output** | **Model** | **Hospital** | **Total negative** | **Predicted as negative** | **TP** | **FP** | **TN** | **FN** |
| --- | --- | --- | --- | --- | --- | --- | --- | --- |
| PO-AKI | DL-IVSS_PCFs 11 | Developmental cohort for threshold | 4815 | 4824 | 92 | 218 | 4597 | 227 |
|  |  | Developmental cohort for test | 4816 | 4809 | 101 | 225 | 4591 | 218 |
|  |  | EVC 1 | 44574 | 44752 | 539 | 1802 | 42772 | 1980 |
|  |  | EVC 2 | 11679 | 11751 | 133 | 374 | 11305 | 446 |
|  | Ensemble_PCFs 11 | Developmental cohort for threshold | 4815 | 4795 | 101 | 238 | 4577 | 218 |
|  |  | Developmental cohort for test | 4816 | 4771 | 120 | 244 | 4572 | 199 |
|  |  | EVC 1 | 44574 | 42871 | 884 | 3338 | 41236 | 1635 |
|  |  | EVC 2 | 11679 | 11386 | 204 | 668 | 11011 | 375 |
| Critical AKI | DL-IVSS_PCFs 11 | Developmental cohort for threshold | 5081 | 4881 | 18 | 235 | 4846 | 35 |
|  |  | Developmental cohort for test | 5082 | 4902 | 20 | 213 | 4869 | 33 |
|  |  | EVC 1 | 46645 | 44885 | 115 | 2093 | 44552 | 333 |
|  |  | EVC 2 | 12147 | 11914 | 28 | 316 | 11831 | 83 |
|  | Ensemble_ PCFs 11 | Developmental cohort for threshold | 5081 | 4905 | 24 | 205 | 4876 | 29 |
|  |  | Developmental cohort for test | 5082 | 4928 | 20 | 187 | 4895 | 33 |
|  |  | EVC 1 | 46645 | 43414 | 196 | 3483 | 43162 | 252 |
|  |  | EVC 2 | 12147 | 11748 | 42 | 468 | 11679 | 69 |

Abbreviations: PO-AKI= Postoperative acute kidney injury; EVC= External validation cohort; TP= True positive; FP= False positive; TN= True negative; FN= False negative; DL-IVSS_PCFs 11= A deep-learning algorithm leveraging time-series intraoperative vital sign signals and preoperative clinical features 11; Ensemble_PCFs 11= A ensemble model combining preOp_ML and DL-IVSS_PCFs 11
