# Supplementary material for: A deep-learning algorithm using real-time collected intraoperative vital sign signals for predicting acute kidney injury after major non-cardiac surgeries: A modelling study
Source: PLoS Med. 2025 Apr 29;22(4):e1004566. doi: 10.1371/journal.pmed.1004566 (PMC12040160; doi:10.1371/journal.pmed.1004566)

**S1 Fig. Detailed data preprocessing and dataset construction flow for the developmental cohort (SNUH)**

(SNUH= Seoul National University Hospital; EHR= Electronic Health Records; DBP= Diastolic blood pressure; SBP= Systolic blood pressure; IDBP= Invasive diastolic blood pressure; ISBP= Invasive systolic blood pressure; HR= Heart rate).


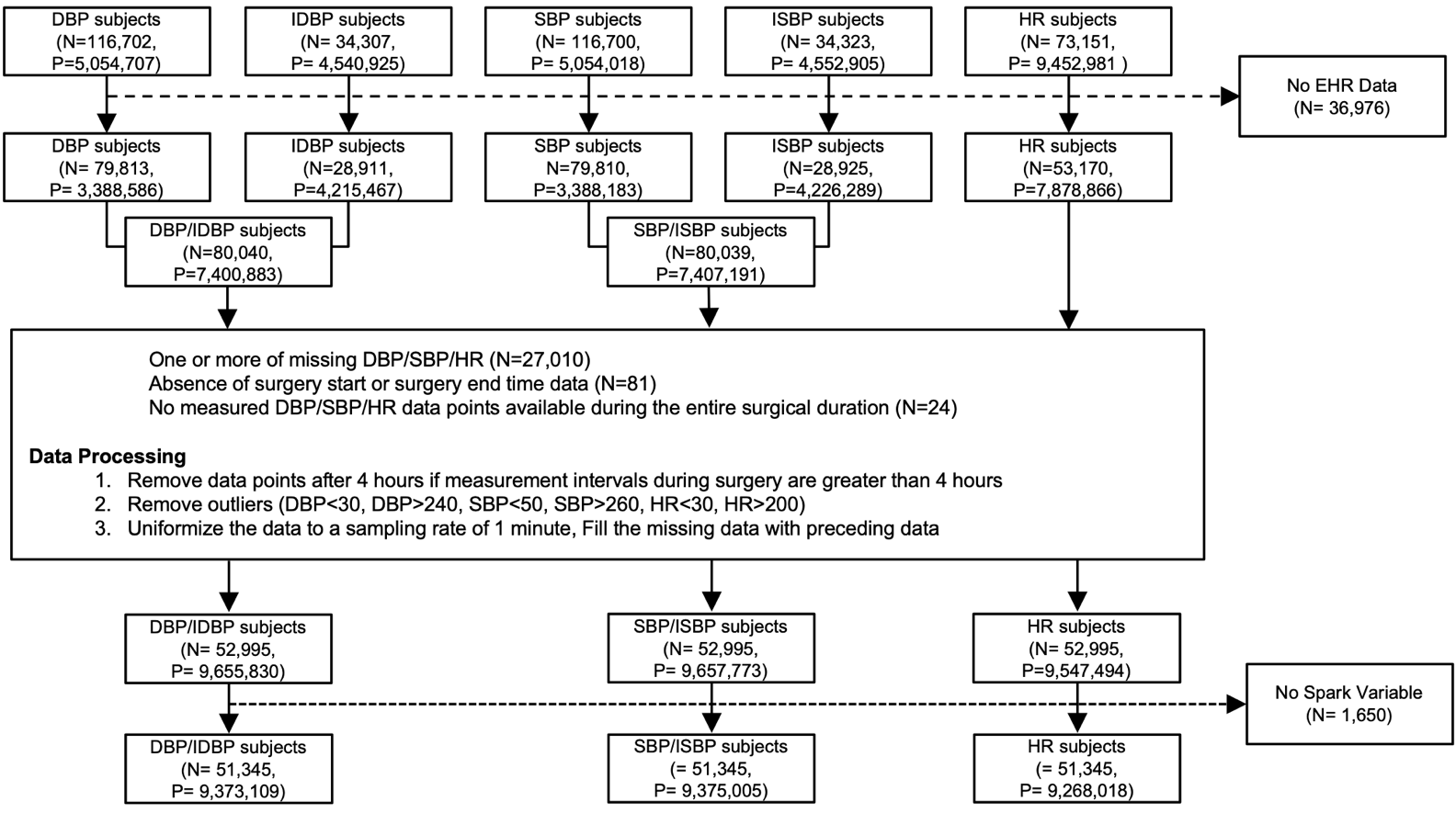

Supplement: S1 Fig — SNUH = Seoul National University Hospital; EHR = Electronic Health Records; DBP = Diastolic blood pressure; SBP = Systolic blood pressure; IDBP = Invasive diastolic blood pressure; ISBP = Invasive systolic blood pressure; HR = Heart rate. (DOCX) [file pmed.1004566.s011.docx]
